# Acoustic Rayleigh Wave Turbulence in Soft Viscoelastic Matter

**DOI:** 10.1002/advs.202407528

**Published:** 2025-02-22

**Authors:** Mikheil Kharbedia, Horacio López‐Menéndez, Basilio Javier García, Manuel G. Velarde, Francisco Monroy

**Affiliations:** ^1^ Departamento de Química Física Universidad Complutense de Madrid Ciudad Universitaria s/n Madrid 28040 Spain; ^2^ Translational Biophysics Instituto de Investigación Sanitaria Hospital Doce de Octubre Avenida Andalucía s/n Madrid 28041 Spain; ^3^ Departamento de Física Aplicada Universidad Autónoma de Madrid Avenida Francisco Tomás y Valiente 7 Madrid 28049 Spain; ^4^ Instituto Pluridisciplinar Universidad Complutense de Madrid Paseo Juan XXIII 1 Madrid 28040 Spain; ^5^ Escuela de Arquitectura Ingeniería y Diseño Universidad Europea de Madrid Villaviciosa de Odon 28670 Spain; ^6^ Present address: Advanced Research Center for Nanolithography (ARCNL) Science Park 106 Amsterdam 1098 XG Netherlands

**Keywords:** acoustic waves, capillary waves, rayleigh waves, soft solids, surface acoustic waves, viscoelastic matter, wave turbulence

## Abstract

Evidence of discrete acoustic Rayleigh wave turbulence (DARWT) is reported on the free surface of complex viscoelastic materials under monochromatic excitation. These surface elastic non‐linear Rayleigh waves exhibit dispersionless dynamics governed by bulk shear rigidity at coexistence with dispersive capillary waves supported by surface tension. Using Laser Doppler Vibrometry (LDV), the discrete Kolmogorov–Zakharov (KZ) spectrum is measured, characterized by a power‐law envelope in an inertial cascade of discrete harmonics. When shear rigidity dominates, KZ‐spectrum characterized by an ω^−5/2^ acoustic fingerprint is observed as a conservative solid‐like scaling, theoretically predicted from the exact three‐wave dispersionless resonance. Conversely, when surface tension dominates over bulk stresses, dispersive CW behavior under the usual ω^−17/6^ liquid‐like scaling is recovered. These findings support a unique theoretical framework for understanding conservative DARWT and advance the field of surface wave turbulence in viscoelastic media with markedly non‐Newtonian rheology. Acoustic Rayleigh wave turbulence represents a conservative mechanism for dispersionless energy transport through weakly nonlinear wave interactions across scales, with relevance to various physics fields, from soft matter and biological systems up to geophysical flows.

## Graphical abstract

Discrete acoustic Rayleigh wave turbulence (DARWT) is observed on the free surface of viscoelastic materials under monochromatic excitation. Governed by bulk shear rigidity supporting inertial modes against surface tension, the nonlinear Rayleigh waves exhibit distinct power‐law scaling and turbulence features in their Kolmogorov–Zakharov spectra. This study provides a unified framework for conservative DARWT, offering insights into surface discrete wave turbulence across diverse physical systems with a supporting viscoelasticity.

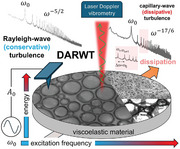

## 1 Introduction

Wave turbulence is a ubiquitous nonlinear phenomenon occurring in nature across scales,^[^ ^1^ ^]^ ranging from astrophysical plasma (e.g., Alfvén waves),^[^ ^2^ ^]^ ordinary “water” waves in classical fluids,^[^ ^3^, ^4^, ^5^ ^]^ to Bose‐Einstein condensates,^[^ ^6^ ^]^ and quantum fluids.^[^ ^7^ ^]^ Wave turbulence kinetically developed is inertia‐dominated, as characterized by frictionless energy flux across different scales.^[^ ^8^ ^]^A conservative (inertial) energy cascade is bounded by the fundamental mode wavenumber *k*_0_, where energy is injected, and the maximum wavenumber *k_K_*, where frictional forces dominate in a terminal Kolmogorov domain.^[^ ^8^, ^9^ ^]^ Turbulent energy cascades occur inviscid within this inertial interval (*k*_0_ < *k* <  *k_K_*), where momentum and energy transfer nonlinearly through resonant couplings $\omega_{1}\left(k_{1}\right)\pm \omega_{2}\left(k_{2}\right)\pm \omega_{3}\left(k_{3}\right)\text{…}\pm \omega_{N}\left(k_{N}\right)=\Omega_{N}$ (with *N* ≥ 3 being the number of interacting waves). Due to finite propagation velocity, wave interactions lead to resonance broadening, $\Omega_{N}\equiv \tau_{NL}^{-1}$, which represents the inverse lifetime of nonlinear energy exchange;^[^ ^10^ ^]^ for long‐lived exact resonance, Ω_*N*_ ≡ 0, while faster scattering interactions result in Ω_*N*_ > 0.^[^ ^1^ ^]^ Nonlinear wave order of any kind can induce *discrete wave turbulence* under exact (or quasi‐exact) resonances (Ω_*N*_≅0).^[^ ^11^, ^12^ ^]^ In viscoelastic matter, for instance, rheological interactions can shape wave dynamics into structured energy cascades modulated by wave dispersivity ω(*k*).^[^ ^13^, ^14^ ^]^ Often, viscoelastic turbulence has been shown organized under rheology‐structure scaling relationships.^[^ ^15^, ^16^, ^17^ ^]^ In incompressible fluids, however, turbulence involves all possible modes leading to unsteady, chaotic flow fully developed―Landau's turbulence.^[^ ^8^ ^]^ Discreteness arises in turbulent wave trains with finite mode spacing, $\Delta_{\omega }\equiv k_{0}^{-1}\partial \omega /\partial k$, which travel faster than coupled mode disentangling may happen, Δ_ω_ ≫ Ω_*N*_ (driven by the fundamental mode *k*_0_, under group velocity ω′ ≡ ∂ω/∂*k*).^[^ ^1^, ^10^, ^11^ ^]^ Other finite‐size effects, including systemic anisotropies or spatial confinements, such as “container” eigenmodes, can support wavefield discreteness, organizing coupling resonances into wave clusters.^[^ ^18^, ^19^ ^]^ Canonical discrete turbulence―as earlier formulated by Karthasova^[^ ^20^ ^]^represents an ordered class of mode coupling that relies on exact resonant clustering, hence featuring integrable high‐dimensional dynamics connecting clustered waves.^[^ ^11^, ^21^ ^]^ In the absence of systemic wave clustering, decoherence effects such as random excitation, wave dispersion, scattering, or simply an infinite container size can elicit the waving manifold to become disordered under resonance broadening greater than mode spacing (Ω_*N*_ ≫ Δ_ω_).^[^ ^21^ ^]^ In a seminal work, Zakharov and Filonenko described random turbulent waves as kinetic wave turbulence,^[^ ^22^ ^]^ marked by strong resonance broadening (Ω_*N*_/ω ≫ 1), appeared as a continuous power spectrum.^[^ ^23^ ^]^ The Kolmogorov–Zakharov (KZ) spectrum of turbulence―a key kinetic feature―follows a scaling power law, $I_{KZ}\left(\omega \right)\;∼\omega^{-\alpha }$,^[^ ^23^ ^]^ reflecting nonlinear wave couplings governed by Hamiltonian symmetries and dimensionality.^[^ ^1^ ^]^ The KZ‐spectrum, characterized by the scaling exponent α, highlights the dispersive nature of the coupled wave modes.^[^ ^1^, ^22^, ^23^ ^]^ As formulated in the classical theory of water waves,^[^ ^23^ ^]^ kinetic surface turbulence involves weakly interacting modes with three‐ or four‐ waves coupling at most.^[^ ^8^ ^]^ Under high wave dispersivity, $\omega ∼k^{\beta }$ (with β > 1, thus Δ_ω_ ≫ 0), random excitation generates disordered liquid assemblies of only primary clusters, essentially triads or quartets, with a homogeneous phase distribution.^[^ ^1^, ^8^, ^21^ ^]^ Most experimental studies on such fluidlike kinetic turbulence have focused on nonlinear capillary waves (CWs) excited on the free surface of Newtonian fluids,^[^ ^1^, ^21^, ^23^ ^]^ driven by the fundamental three‐waves interaction locked into sister triads (i.e., ω_1_ + ω_2_⇒ω_3_).^[^ ^24^, ^25^, ^26^ ^]^ Capillary “water” waves propagate under surface tension (σ), following liquidlike dispersion, ω_*CW*_ ≈ (σ*k* ^3^/ρ)^1/2^, termed decay‐type as shorter ripples travel faster in wavefront trains than the wave‐packet as a whole (the phase velocity $c_{CW}\equiv \omega /k∼k^{1/2}$, then $\omega_{CW}^{\prime }=\beta_{CW}\omega /k>c_{CW}$).^[^ ^10^ ^]^ Furthermore, Hamiltonian symmetries in convex propagation subspaces (ω′′ ≡ *d* ^2^ω/*dk* ^2^ ≥ 0 for β ≥ 1), enable quasi‐exact resonances to essentially govern capillary cascades of developed turbulence upon high dispersivity (β_*CW*_ = 3/2).^[^ ^8^ ^]^

Acoustic, solidlike wave turbulence in materials with shear rigidity (*G*) has received less attention,^[17]^ despite nonlinear elasticity wavefields are supported without dispersivity, ω ≈ *ck* (at constant velocity, *c* ≈ (*G*/ρ)^1/2^ ≈ ω′, under collinear coupling, ω′′ = 0, upon β = 1).^[^ ^15^, ^27^, ^28^ ^]^ Hence, acoustic waves could be discretely excited dispersionless as 2D sound on solid interfaces, eventually leading to nonlinear density perturbations known as *elastic wave turbulence*.^[^ ^15^, ^16^, ^17^, ^28^ ^]^ Indeed, three‐wave interactions are the primary couplings in elastic media, resulting in exact resonances (Ω_*N* = 3_ = 0).^[^ ^29^ ^]^ However, scattering can disrupt triadic resonances^[^ ^30^ ^]^ making higher‐order interactions—such as scattering‐adapted quartets (ω_1_ + ω_2_⇒ω_3_ + ω_4_ + Ω_*N* = 4_), more relevant in systems with non‐decaying dispersivity (Ω_*N* = 4_ > 0 with β < 1).^[^ ^23^ ^]^ Although exact four‐wave interactions are always possible in isotropic media,^[^ ^1^, ^23^ ^]^ they request on strict collinear alignment achievable only in the quasi‐elastic scattering limit (*k* → 0), under non‐decaying concave dispersion (ω′′ < 0),^[^ ^30^ ^]^ as seen in gravity water waves (upon non‐decaying dispersivity β_*GW*_ = 1/2).^[^ ^1^ ^]^ Relevantly, surface pure‐elastic turbulence has been observed from nonlinear bending waves excited on vibrating flexible plates.^[^ ^31^, ^32^ ^]^ However, no mode discretization was detected in those studies revealing a continuous KZ‐spectrum.^[^ ^31^, ^32^ ^]^ Even under monochromatic excitation, multiple scattering disrupted wave coherence resulting in significant resonance broadening (Ω_*N*_ ≫ 0).^[^ ^32^ ^]^ Besides, the inertial interval was observed to be pathologically narrow (less than one frequency decade long), with spectral features deviating significantly from theoretical predictions.^[^ ^32^ ^]^ Although the continuous KZ‐spectrum aligned with the elastic‐like energy scaling expected from weak three‐wave coupling, its frequency dependence was closer to four‐wave predictions. Finally, the frictional shutdown was too abrupt to match Kolmogorov scaling, as expected in the smallest scales of observed turbulence.^[^ ^8^ ^]^ While most studies have described elasticity‐driven acoustic turbulence,^[^ ^14^, ^15^, ^16^, ^17^, ^33^, ^34^ ^]^ its Hamiltonian nature in discrete wave ensembles remains unresolved due to limited experimental evidence. Whether conservative elastic turbulence occurs through surface acoustic waves within bulky viscoelastic media remains indeed a matter of conjecture. Henceforth, we aim to experimentally confirm that, in discrete surface wavefields supported by bulk rigidity, exact three‐wave acoustic resonances dominate (i.e., Δ_ω_ ≫ Ω_*N* = 3_ → 0 for *N* = 3), over higher‐order couplings leading multiple scattering under dispersive non‐exactness (Ω_*N* > 3_ > Δ_ω_ ≫ 0 for *N* > 3). To study discrete turbulent modes under nonlinear surface wave couplings observable in KZ spectra, we will investigate a wide range of viscoelastic regimes—from fluidlike systems with decay‐type dispersion (β > 1, ω′′ > 0, thus Ω_*N*_ > Δ_ω_), to solidlike systems with restricted to 2D acoustic, dispersionless propagation (β = 1, ω′′ = 0, thus Ω_*N*_ → 0).

In this work, we examine nonlinear Rayleigh waves (RWs)—dispersionless surface acoustic waves that propagate efficiently on soft solids due to bulk shear rigidity.^[^ ^35^ ^]^ Their solidlike bulk elasticity should impart surface wave order promoting discretization and resonance in acoustic wave turbulence. By reducing dispersivity (cf. scattering), turbulent RW energy cascades are expected to extend over broader inertial intervals than CWs. Surface wave theory predicts both, dissipative CWs and non‐dispersive acoustic waves.^[^ ^10^ ^]^ Particularly, RWs propagate dispersionless on soft viscoelastic solids without energy loss via a purely elastic Kelvin–Voigt response.^[^ ^35^, ^36^ ^]^ Previous experimental studies have confirmed the coexistence of these surface waves on soft hydrogels, where high surface tension and bulk rigidity jointly influence enhanced wave propagation.^[^ ^37^, ^38^ ^]^ On the theoretical side, emergent (RW‐driven) nonlinearities have been emphasized, such as convective transport by surface modes and their coupling interactions arising from the viscoelastic character.^[^ ^39^ ^]^Notably, “supershear” RWs with unusually high velocities have been observed on solidlike viscoelastic surfaces.^[^ ^40^ ^]^ However, no evidence of nonlinear RW turbulence has yet been reported, either in the kinetic regime for energy conservation or under conditions that allow exact resonances with the driving source.

We focus on discrete acoustic Rayleigh wave turbulence (DARWT), developed as nonlinear Rayleigh waves (RWs) propagating dispersionless on soft solid surfaces. DARWT inertial cascades were observed in the solidlike regime falling from a monochromatic source down to a disordered Kolmogorov domain ending in frictional dissipation. Experiments were performed on swollen hydrogels, water‐in‐oil emulsions, and dry foams chosen as viscoelastic materials with surface tension (σ) and bulk viscosity (η), supporting Rayleigh waves under sufficient bulk rigidity (*G* ≫ ωη, being ω the wave frequency). Using laser Doppler velocimetry,^[^ ^41^ ^]^ we examined nonlinear RW coupling, exact resonances, and turbulence scaling at inertial intervals, highlighting spectral narrowing under minimal scattering. DARWT coherence is revealed maintained despite dissipative effects in various viscoelastic solids, from isotropic gels and emulsions to inhomogeneous foams. Insights from this work apply to soft solids e.g., polymers, liquid crystals, and cellular materials, offering a framework to model nonlinear viscoelasticity in fields such as geophysics, materials science, plasma technology, and biomedical engineering.^[^ ^42^, ^43^, ^44^ ^]^

## 2 Experimental Section

### 2.1 Agarose Hydrogels: Soft‐Solid Rheology

To characterize surface Rayleigh wave (RW) dynamics, swollen agarose hydrogels were used for the viscoelastic properties, previously investigated in detail.^[^ ^35^ ^]^ Agarose powder (Sigma–Aldrich) was dissolved in Milli‐Q water, heated to 80 °C while shaken with a vortex generator, then cooled to room temperature for 1 h to form a polymeric hydrogel. The agarose gels were known to fulfill the rheological premises of the HPP theory for RWs.^[^ ^35^, ^36^ ^]^ As soft viscoelastic continua, they were described by the Kelvin–Voigt model with constitutive relations of the type η(ω) =  η_0_ + *G*/*i*ω,^[^ ^36^, ^37^, ^38^ ^]^ corresponding to a linear viscoelastic response under constant bulk rigidity (*G*) (Figure S1, Supporting Information). Here, η(ω) is a complex frequency‐dependent viscosity, with η_0_ denoting the reference value of the Newtonian embedding (water solvent), and *G* is the frequency‐independent rigidity modulus of the polymeric network (see Note S1 and Figure S2, Supporting Information). In addition to physically entangled agarose hydrogels, various other types of viscoelastic materials, chemically crosslinked polymer gels, solid foams, and liquid w/o‐emulsions, for instance, were considered to assess the universality of the RW spectrum of turbulence, encompassing structural and dynamic scales spanning several orders of magnitude (Figure S3, Supporting Information). The viscoelastic response of these soft solid materials i.e., *G*(ω) and η(ω) were measured with an oscillatory rheometer (Hybrid Rheometer, TA Instruments). Two different rheological measurements were performed: *1) Amplitude Sweep*: the material was sheared at a constant frequency (1 Hz) with varying amplitude (0%–10,000%), to establish the linear and nonlinear regime of viscoelastic response to dynamic shear stress. *2) Frequency Sweep*: the dynamic response was measured at a variable frequency (0.1–100Hz) under constant shear strain (1%) (see Figures S1,S2, Supporting Information for detailed explanations). The surface tension was measured with respect to the bare water surface (σ_0_ ≈ 0.072 *N*/*m*), and the bulk density considered constant (ρ ≈ 10^3^ *kg*/*m* ^3^). Rheological measurements were conducted on various viscoelastic materials to cover a range of soft solid behavior (Note S2, Supporting Information).

### 2.2 Surface Wave Excitation

To study surface WT without distorting and /or confinement of the excited wavefield, the hydrogel was placed in a circular container with a diameter *D* = 15*cm* and a depth *h* = 2*cm*. This size ensures large‐box conditions for typical RW wavelengths around λ = 2*mm* (*D* ≫ λ). With preserving short wavelengths relative to vessel depth (λ ≪ *h*), the RW amplitudes were expected to exhibit exponential decay into the material bulk.^[^ ^35^ ^]^ Hence, reflectance or interference was minimized with the container boundaries. The surface waves were excited using a transverse shearing blade attached to a mechanical vibrator (Brüel & Kjaer 4809), controlled by a signal generator producing a monochromatic signal (Agilent 33210A). Some experiments used a homemade dichromatic driving system providing a setting for sum‐frequency harmonic excitation with prime numbered modes leading to unambiguously combined sister triads.^[^ ^11^ ^]^ For those experiments using simultaneous double‐frequency primed excitation, sinusoidal voltage signals supplied from two function generators were superposed using an operational amplifier‐based voltage adder. The wave phase velocity was determined using a high‐speed camera vertically focused on the hydrogel surface (see Figure S4, Supporting Information for details).

### 2.3 Velocity Wave Amplitude and Spectral Analysis

To measure the kinetic energy spectra, a Laser Doppler Vibrometer (LDV100, Polytec) mounted vertically on an optical table was utilized (Newport) to accurately detect surface wave propagation.^[^ ^41^ ^]^ A laser 623 nm beam was focused at a single point on the oscillating surface, specifically at the vessel center, to avoid wave distortion from the walls. The laser beam, with a power of 1 mW and a diameter of 2–10 mm, had negligible structural and thermal impact. No physical damage, such as cracking or melting, was observed on the hydrogel after hours of experimentation (see Figure S4, Supporting Information). This LDV device measures the phase shift between incident and reflected waves on the oscillating surface, providing vertical surface velocity data. This technique allowed to explore vertical velocities from 0.1 mm s^−1^ upto 50 m s^−1^, within the 20 kHz acoustic band. The LDV signal was digitized and converted into a time series of velocities, *v*(*t*). Fast Fourier Transform (FFT) was used to calculate power spectral densities in the frequency domain, $PSD\left(\omega \right)=\int v^{2}\left(t\right)e^{-i\omega t}\;dt\Leftrightarrow I_{\omega }$, corresponding to the velocity spectrum (*I*_ω_), representing the kinetic energy of the wavefield. To ensure reproducibility and reliability, each spectrum was averaged over 100 different measurements in at least 3‐4 replicas. The velocity PSDs were then processed to obtain KZ‐spectra under different experimental conditions.

## 3 Theory

### 3.1 Surface Waves in Soft Viscoelastic Materials

In soft gelly structures with a uniform composition in 3D, the HPP theory predicts several types of surface waves both CWs and RWs. For a Kelvin‐Voigt model, these surface modes can be described by the following dispersion equation:^[^ ^36^ ^]^

(1) $$\begin{array}{c} D\left(k,\omega \right)={\left[i\omega +\frac{2\eta \left(\omega \right)k^{2}}{\rho }\right]}^{2}-\\ 4\eta \left(\omega \right)2k^{4}{\left[1+\frac{i\omega \rho }{\eta \left(\omega \right)k^{2}}\right]}^{1/2}+\frac{\sigma k^{3}}{\rho } \end{array}$$

where the frequency‐dependent complex viscosity is denoted by η(ω) = η(ω) =  η_0_ + *G*/*i*ω, while ρ represents the density of the medium, and σ stands for surface tension.

The surface wave dispersion relation ω(*k*), which relates wave frequency to wavevector (*k*), can be determined by solving *D*(ω, *k*) = 0. In genuine fluid scenarios, such as when surface tension dominates over bulk rigidity i.e., σ*k* ^3^ > η(ω)^2^ *k* ^4^, surface flexural wave propagation follows the expected CW‐dispersion as the Kelvin relationship:

(2) $$\omega_{C}^{2}\approx \frac{\sigma k^{3}}{\rho }$$

In soft solids, when the shear modulus (*G*) prevails over surface tension (*G* ^2^ *k* ^4^/ω^2^ > σ*k* ^3^), and surpasses the viscosity of the medium (*G* > ωη_0_), the mechanical coupling between surface and bulk stresses originates surface Rayleigh waves (RW) obeying the expected non‐dispersive relation of acoustic waves:

(3) $$\omega_{R}^{2}\approx \frac{Gk^{2}}{\rho }$$

The HPP theory also predicts a non‐propagating overdamped wave (when η_0_ ≫  *G*/ω), which is not relevant in this context. A common method to distinguish between these two types of waves is by measuring their phase velocity *c* = ω/*k*, as they propagate on soft surfaces (refer to Figures S4,S5, Supporting Information). According to the dispersion relations given in Equations (2), (3), for CW and RW, one obtains respectively:

(4) $$\begin{array}{cc} c_{CW}\approx {\left(\frac{\sigma \omega }{\rho }\right)}^{1/3}, & c_{RW}\approx {\left(\frac{G}{\rho }\right)}^{1/2} \end{array}$$

**Figure 1:**
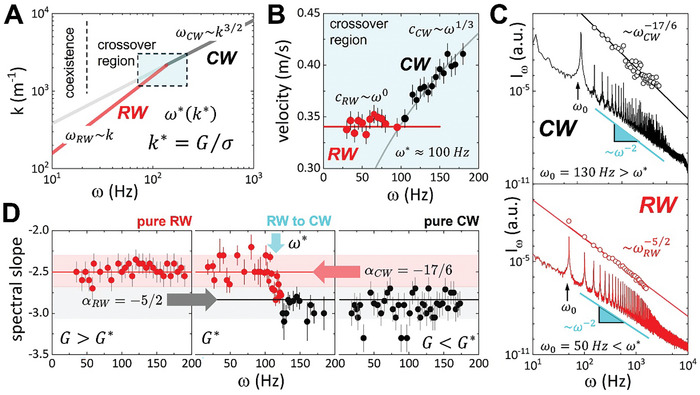
Surface waves on viscoelastic matter. Generic propagation features. A) Three types of surface waves are predicted by the HPP theory: Non‐dispersive Rayleigh waves (RWs) propagate acoustically as ω_*RW*_  ≈ (*G*/ρ)^1/2^ *k* (red line; *G* is the shear rigidity), coexisting with capillary waves (CWs) at low frequencies (gray lines). Dispersive CWs, characterized by $\omega_{CW}^{2}\;\approx \sigma k^{3}/\rho$(σ is the surface tension and ρ the bulk density), persist at high frequency. At very high rates, friction‐overdamped non‐propagating waves appear as $\omega_{D}\;∼\;i\left(\eta /\rho \right)k^{2}$ (η is the shear rigidity; not shown). A crossover occurs where RW and CW propagation equalize (ω_*CW*_ = ω_*RW*_ = ω*), defining critical conditions k* = *G*/σ, thus ω* = (*G* ^3^/ρσ^2^)^1/2^. In the crossover ω* − k*, this transition spans RW/CW coexistence at low frequencies to pure CW dominance at high frequencies (see main text). B) Propagation phase velocity regimes on the agarose hydrogel at the crossover point (systemic parameters: G* = 80 *Pa*,  σ = 0.072 *N*/*m* and ρ = 10^3^ *kg*/*m* ^3^). RWs under slow acoustic propagation at constant velocity *c_RW_*  ≈  (*G*/ρ)^1/2^ (at ω_*R*_ < ω*; red symbols). CWs under faster dispersivity with increasing velocity *c_CW_*  ≈  (σω/ρ)^1/3^  ≫ *c_RW_* (at ω_*C*_ > ω*; black symbols). Straight lines represent theoretical predictions from systemic material parameters (RW: red; CW: grey). The RW/CW crossover is found at ω* ≈ 100 *Hz*, as predicted from systemic parameters. C) KZ‐spectra of discrete turbulence excited at variable excitation frequency (ω_0_). The nonlinear surface waves are excited in the agarose gel in the different propagation regimes relative to crossover frequency (for *G* = 80 *Pa* > G*); CW‐like spectrum (ω_0_ = 130*Hz* > ω*; upper panel); RW‐like spectrum (lower panel; ω_0_ = 50*Hz* < ω*). Both KZ‐spectra exhibit direct cascades of discrete harmonics with scaling frequency decay characterized by different power laws; for CWs following. *I*_ω_∝ω^−17/6^ and for RW as *I*_ω_∝ω^−5/2^. The open symbols over spectral peaks represent discrete harmonic amplitudes, with the best‐fitting slope (scaling exponent) based on the corresponding power law. D) KZ‐spectral slopes, calculated in the RW/CW excitation range (ω_0_ ≈ 20 − 200*Hz*), for three different hydrogels with a variable shear rigidity (from left to right): pure RW regime (*G* ≈ 1.5 *kPa* ≫ G*); crossover region from RW to CW (*G* ≈ G* ≈ 80 *Pa*); pure CW regime (*G* ≈ 6 *Pa* ≪ G*). The straight lines represent the best fittings KZ‐spectra. *I*_ω_∝ω^−α^, with scaling exponent either for capillary wave turbulence (α_*CW*_ = 17/6 at ω_0_ > ω*), or for discrete acoustic Rayleigh wave turbulence (DARWT) revealed at low frequencies (α_*RW*_ = 5/2 at ω_0_ < ω*). The spectral background represents developed fluid turbulence as a Landau's continuum (*I_back_* ∝ω^−2^; cyan); see main text for details.

We must emphasize that the phase velocity of CWs depends on frequency (dispersive ripples dependent on σ), whereas RWs propagate dispersionless at a constant phase velocity solely determined by bulk rigidity (*G*). **Figure** **1**A illustrates the surface wave diagram in terms of frequency ω(*k*), for apparent complex elasticity, *G*(*k*) = *i*ω(*k*)η, both being functions of wavevector in soft solids. The crossover point (G*, k*) between different CW/RW types corresponds to G* = ρ(σ/η_0_)^2^, and $k^{*}=\rho \sigma /\eta_{0}^{2}$ with $\omega^{*}={\left(G\rho \right)}^{1/2}\left(\sigma /\eta_{0}^{2}\right)$.^[^ ^36^ ^]^ With a relatively low shear viscosity (e.g., η_0_ ≈ 0.1 −10 *Pa*.*s* for swollen hydrogels), surface wave excitation can be controlled by adjusting the bulk modulus (*G* ≥ ωη_0_), exploring both CW and RW regimes, determining their phase velocities, and studying linear and nonlinear spectra. Increasing the bulk modulus (for *G* > G*), at a constant excitation frequency ω, transitions from CW to RW can be modulated. Conversely, at given bulk rigidity, transitioning from RW to CW occurs by increasing excitation frequency (at ω > ω*) (Figure S5, Supporting Information).

### 3.2 Kinetic Wave Turbulence

As described by the classical ZF‐theory,^[^ ^7^, ^8^, ^9^ ^]^ the random dynamics of weakly interacting turbulent ensembles induced by kinetic energy injection is usually described by a wavefield Hamiltonian (*H* = *H*_2_ + *H_i_*), composed of linear (*H*_2_), and higher‐order perturbative terms (*H_i_*, with i=3,4,…), indicating increasing weak nonlinearity with a reduced number of interacting waves contributing to energy cascade across different scales.^[^ ^1^, ^23^ ^]^ In the limit of weak turbulence, the three‐wave coupling interaction holds dominant *H*_2_ > *H*_3_ ≫ *H*_4_ (thus *N* = 3).^[^ ^23^ ^]^ An appropriate statistical description of kinetic turbulent behavior relies on time correlation functions of energy amplitudes; hence, the wave population distribution is presented as:

(5) $$n_{k}\equiv \langle a\left(k,t\right)a{\left(k,t\right)}^{*}\rangle$$

where *a*(*k*, *t*) is the complex wave amplitude and *a*(*k*, *t*)* its corresponding complex conjugate. Based on the first principle that the energy cascade is a function of the energy flux toward different scales, dimensional analysis leads to the generalized scaling law for the mode populations:^[^ ^1^, ^23^ ^]^

(6) $$n_{k}∼\omega_{k}^{\left(N-4\right)/\left(N-1\right)}\varepsilon^{1/\left(N-1\right)}k^{\left(10-d-5N\right)/\left(N-1\right)}$$

In this dimensional formula, ω_*k*_ accounts for dispersion law, *k* denotes wavenumber, $\varepsilon \equiv {\dot{E}}_{k}$ is the injected power, *N* is the number of interacting waves satisfying energy conservation, and *d* is the spatial dimension relevant to wave propagation. The number *N* can be determined based on symmetry arguments of the dispersion relation.^[^ ^1^, ^10^, ^23^ ^]^ When the constitutive dispersion relation $\omega \;∼\;k^{\beta }$ follows dispersive, decay‐type propagation (β > 1), or non‐dispersive propagation (β = 1), a fundamental three‐wave interaction is permissible (*N* = 3). As described by Zakharov and Filonenko (ZF),^[^ ^8^ ^]^ this three‐wave dispersive condition defines kinetic turbulence with nonlinear CWs, where β_*CW*_ = 3/2.^[^ ^23^ ^]^ For backward retarding modes (β < 1), exact three‐wave interactions are forbidden, resulting in higher nonlinearity through four‐wave interactions (*N* = 4), under quasi‐exact dismutation resonance, as seen in gravity waves (GWs), with β_*GW*_ = 1/2.^[^ ^1^, ^21^ ^]^

#### 3.2.1 Kolmogorov–Zakharov (KZ) Spectrum

In the context of kinetic wave turbulence,^[^ ^1^ ^]^ it is practical to analyze the autocorrelation function of vertical wavefield displacement A(r,t), which is defined as:^[^ ^23^ ^]^

(7) $$\begin{array}{c} I_{\omega }\equiv \langle {\left|A\right|}^{2}\rangle =\\ \left(\frac{1}{2\pi }\right)\int A\left(r,t\right)A\left(r,t+\tau \right)e^{-i\omega t}d\tau . \end{array}$$

Hereafter, our results will be presented in terms of the spectral density *I*_ω_(ε), defined as a function of the injection rate of kinetic energy (ε). From dimensional analisys, the power‐dependent spectral function can be expressed in terms of wavenumber (*k*), in reciprocal spatial *k* − space:^[^ ^1^ ^]^

(8) $$I_{k}\left(\varepsilon ,k\right)=\frac{dk}{d\omega }n_{k}\left(\varepsilon \right)\frac{k^{d}}{\omega_{k}}$$

or in temporal frequency ω − space:^[^ ^8^ ^]^

(9) $$I_{\omega }\left(\varepsilon ,\omega \right)=\frac{dk}{d\omega }n_{\omega }\left(\varepsilon \right)$$

where the choice between the manifold populations, *n_k_* (ε), *n*_ω_(ε), depends on the dispersion relation used. From Equation (9), we derive the KZ‐spectrum of kinetic turbulence characterized by the power law *I*_ω_∝ω^−α^, where α is the KZ‐exponent for spectral distribution specific to the surface waves under consideration.^[^ ^1^, ^21^ ^]^

#### 3.2.2 The Canonical Case of Discrete Capillary Wave Turbulence (DCWT)

Capillarity‐dominated surface waves follow the CW‐dispersion relation $\omega ∼k^{\beta }$ (with  β_*CW*_ = 3/2; refer to Kelvin's formula in Equation 2). Thus, leading nonlinearity is three‐waves coupling (*N* = 3), reflecting their fundamentally interfacial nature (*d* = 2). Subsequently, applying Equation (9) the well‐established KZ‐spectrum of CW‐turbulence was derived as the well‐known ZF‐formula for canonical three‐wave turbulence in 2D:^[^ ^8^ ^]^

(10) $${\left.I_{\omega }\right|}_{CW}^{2D}\propto \varepsilon^{1/2}{\left(\frac{\sigma }{\rho }\right)}^{1/6}\omega^{-17/6}$$

which has been extensively discussed in the literature.^[^ ^1^, ^23^ ^]^

#### 3.2.3 The New Case of Discrete Acoustic Rayleigh Wave Turbulence (DARWT)

When bulk shear rigidity dominates the surface wavefield, as observed in soft viscoelastic materials,^[^ ^36^, ^37^, ^38^ ^]^ discrete acoustic Rayleigh wave turbulence (DARWT) is expected to emerge within a specific range of excitation frequencies (ω_*k*_), from the injection mode (ω_0_) up to a maximum Kolmogorov frequency (ω_*max*_≅ω_*K*_). Their acoustic propagation is described by ω_*k*_  ≈ (*G*/ρ)^1/2^ *k* (with β_*RW*_ = 1; Equation 3), under exact three‐wave coupling (*N* = 3) as the leading nonlinearity.^[^ ^29^ ^]^ Considering the dominant influence of the bulky solid properties, the relevant 3D are considered (*d* = 3).^[^ ^35^ ^]^ Hence, we obtain the KZ‐spectrum for canonical DARWT with a power‐law scaling defined in 3D:

(11) $${\left.I_{\omega }\right|}_{RW}^{3D}\propto {\left(\varepsilon \rho \right)}^{1/2}\omega^{-5/2}$$

under unique RW‐ exponent (α_*RW*_ = 5/2), distinct from CWs (α_*CW*_ = 17/6), yet sharing a similar kinetic dependence on injected power ($∼\varepsilon^{1/2}$). This new formula serves as a reliable KZ‐spectrum for nonlinear Rayleigh waves generating acoustic turbulence on the surface of solids.

Whether acoustic turbulence becomes confined to interfacial elasticity without significant impact on the bulk properties of the soft solid material the nonlinear wave dynamics scaling would be noteworthy 2D (*d* = 2) e.g., with viscoelastic layers on liquid surfaces,^[^ ^44^ ^]^ strictly 2D foams,^[^ ^45^ ^]^ or liquid layers with surfactant‐induced Marangoni effect.^[^ ^46^, ^47^ ^]^ In such cases, the corresponding turbulence KZ‐spectrum in 2D would be:

(12) $${\left.I_{\omega }\right|}_{RW}^{2D}\propto {\left(\varepsilon \rho \right)}^{1/2}\omega^{-2}$$

which exhibits a very distinctive quadratic decay ($∼\omega^{-2}$), to be further identified in experiments with viscoelastic surface layers.^[^ ^48^ ^]^ However, in our current study where non‐dispersive RW refers to bulky soft solids, we adopt 3D (*d* = 3) and refer to Equation (11) for our findings.

### 3.3 Nonlinear Interaction Rate

A fundamental characteristic of the KZ‐spectrum of turbulence is the energy cascade occurring within the inertial interval, which spans various scales where nonlinear mode coupling takes place during a finite interaction time (τ_*NL*_).^[^ ^3^, ^11^, ^23^ ^]^ In the case of a direct cascade (where kinetic energy transfers occur from larger to smaller scales), this inertial interval is delimited by the fundamental frequency ω_0_, and the maximum Kolmogorov frequency, $\omega_{max}\approx \omega_{K}\left(k_{K}\right)≅\tau_{NL}^{-1}$, where the nonlinear interaction rate equals the viscous damping rate, $\tau_{\eta }^{-1}$, hence causing the kinetic energy to be dissipated by friction under injected power $\varepsilon \approx E_{k}/\tau_{NL}\approx V_{k}^{2}/\tau_{NL}$.^[^ ^23^ ^]^ Hence, the nonlinear interaction rate can be approximated as $\tau_{NL}^{-1}\left(\varepsilon \right)≅V_{k}^{2}n_{k}k^{2}/\omega_{k},$where the turbulent kinetic energy $E_{k}\approx V_{k}^{2}\Rightarrow H_{k}^{2}$ promotes the *N*‐wave interaction as described in terms of dispersivity $\omega_{k}∼k^{\beta }$.^[^ ^23^ ^]^ From dimensional analysis, Zakharov deduced *H_k_* ≈ ω_*k*_ *k* ^1/2(5 + β − *d*)^.^[^ ^1^, ^23^ ^]^ For surface acoustic waves ($\omega_{k}∼ck$, thus β = 1, at *d* = 2), when translating these formulas to the frequency domain (Equation 9), the nonlinear interaction rate can be thus expressed as:

(13) $$\tau_{NL}^{-1}\left(\varepsilon \right)\approx V_{\omega }^{2}n_{\omega }\approx A^{2}\omega^{2}n_{\omega }$$

Using this kinetic parameter (*A* is the wavefield amplitude), we define the kinetic condition for exact three‐wave resonance to happen in the inertial DARWT regime (see Note S1, Supporting Information). DARWT begins at a critical amplitude for developed turbulence (*A* ≥ *A_c_*), at the RW fundamental frequency (ω_0_ ≡ *c_RW_k*_0_), determined by the lowest pumping wavevector *k*_0_(ω_0_), under constant acoustic velocity, *c_RW_* ≈ (*G*/ρ)^1/2^. Above this onset (*A* > *A_c_* and ω_0_ > ω*), the RW manifold becomes steadily interactive ($\tau_{NL}^{-1}\left(\varepsilon >\varepsilon_{c}\right)\gg \omega_{0}>\omega^{*}$), enabling discrete acoustic resonances to become stable through exact three‐wave couplings provided sufficient rigidity to overcome faster capillary dispersion (with *G* > σ*k*_0_, within *c_CW_* ≈ (σ*k*_0_/ρ)^1/2^ > *c_RW_*).

### 3.4 Finite Size Discretization and Spectral Broadening

Due to the finite size effects under wave excitation, the clear distinction between discrete integrable and kinetic statistical wave turbulence hinges on comparing the interaction rate of the nonlinearly coupled waves $\tau_{NL}^{-1}\Rightarrow \Omega_{N}$, which leads to resonance broadening, with their systemic spectral broadening $\Delta_{\omega }\approx \frac{2\pi }{L}\frac{d\omega_{k}}{dk}$, where *L* represents the finite system length.^[^ ^49^ ^]^ In wave manifolds confined to a finite vessel or under discrete nonlinear excitation, such as resonant clusters in discrete turbulence,^[^ ^11^, ^19^ ^]^ spectral broadening is determined by the frequency spacing of natural eigenmodes (being *L* the characteristic length of the vessel, for instance). Therefore, when $\tau_{NL}^{-1}\ll \Delta_{\omega }$, finite discretization effects become apparent, allowing for the exploration of the discreteness regime under nonlinear wave excitation. This condition is met only when the excitation of the wavefield is sufficiently strong to surpass the linear response but not intense enough to induce dispersive wave breaking, multiple scattering, or other nonlinear effects beyond the scope of ZF‐theory adapted to discrete resonances.^[^ ^50^ ^]^ Given the experimental setup described and the discreteness relation $\tau_{NL}^{-1}<\omega_{0}\ll \Delta_{\omega }$ (Note S3, Supporting Information), our focus now shifts to investigating discrete turbulence with associated kinetic features due to non‐dispersivity with surface nonlinear RWs (i.e., DARWT) and examining key aspects of weak wave turbulence spectra, both theoretically and experimentally.

### 3.5 Inertial Interval: Upper Kolmogorov Frequency

To analyze the frequency span of the KZ‐spectrum (inertial interval from ω_0_ to ω_*max*_) as a function of kinetic energy input (ε ≡ *E_k_* (*A*)/τ_*NL*_), we relate the Kolmogorov frequency (ω_*K*_ ≈ ω_*max*_) to the amplitude of wave excitation (*A*): the higher the amplitude, the higher ω_*max*_(*A*), and the larger the inertial interval.^[^ ^51^, ^52^, ^53^ ^]^Due to kinetic turbulence dominance, *E_k_* (*A*)≅ρ*V* ^2^/2 ≈ ρ*A* ^2^ω^2^/2 (with wavefield vertical velocity *V* = *A*ω), it is also well‐established that the WT strength can be expressed quadratically as *I*_ω_(*A*) ∼ *n*_ω_ *dk*/*d*ω ∼ *A* ^2^(ω/ω_0_)^α^, where ω_0_ is the fundamental excitation frequency and α is the spectral KZ‐decay exponent.^[^ ^51^ ^]^ Considering Equations (8)–(13) and focusing on the relevant non‐dispersive RWs, we derive: i) the nonlinear interaction rate $\tau_{NL}^{-1}{|}_{RW}^{3D}\approx \left(\rho /G\right)A^{2}{\left(\omega /\omega_{0}\right)}^{-7/2}\omega^{3}$; ii) the viscous damping rate $\tau_{\eta }^{-1}{|}_{RW}^{3D}\approx 2\left(\eta_{0}/G\right)\omega^{2}$.^[^ ^36^ ^]^ As previously noted (Note S3, Supporting Information), both rates become comparable beyond the Kolmogorov frequency ω_*max*_; by setting $\tau_{NL}^{-1}=\tau_{\eta }^{-1}$, and after algebraic manipulation, we obtain the upper boundary of the inertial DARWT interval i.e., the Kolmogorov frequency for nonlinear Rayleigh waves, ω_*max*_, related to the injection lower frequency, ω_0_, and the amplitude of wave excitation, *A*, as follows:

(14) $${\left.\omega_{max}\right|}_{RW}^{3D}≅K_{RW}A^{4/5}\omega_{0}^{7/5}$$

where *K_RW_* ≡ (ρ/2η)^2/5^ is a constitutive constant fixed by the inverse kinematic viscosity i.e., the higher viscosity, the lower Kolmogorov frequency for Rayleigh waves, thus limiting the DARWT‐inertial interval.

A similar analysis for surface CWs yielded the well‐known expression:^[^ ^1^, ^8^, ^10^, ^49^ ^]^

(15) $${\left.\omega_{max}\right|}_{CW}^{2D}≅K_{CW}A^{4/3}\omega_{0}^{23/9}$$

Our experimental work systematically explores the predicted characteristics of the KZ‐spectrum of elastic turbulence with Rayleigh waves (RWs) excited on soft solids. Here, $I_{\omega }{|}_{RW}^{3D}\;∼\;\omega^{-5/2}$ (for ω < ω_*max*_), reflecting a characteristic RW‐scaling decay within a quite broad inertial interval ${\omega_{max}|}_{RW}\;∼\;\omega_{0}^{7/5}$. Indeed, we predict a broader inertial interval for nondispersive RWs, with a weaker spectral decay than found for dispersive CWs. These spectral features illustrate scaling differences due to dispersivity changes within the three‐wave resonance at the genesis of kinetic wave turbulence. We will examine the distinctive KZ‐spectra of the acoustic Rayleigh wave turbulence excited across various materials with different physical properties, hence confirming the universality class of weak elastic DARWT (with dispersionless RWs propagating on soft solids; *N* = 3 and *d* = 3), as predicted from the ZF‐theory.^[^ ^1^, ^23^ ^]^

## 4 Experimental Results

### 4.1 Discrete Nonlinear RWs Excited on Agarose Gels

Figure 1 outlines the experimental rationale for modulated RW/CW propagation on the free surface of an agarose hydrogel considered a model soft solid.^[^ ^36^, ^37^ ^]^These agarose hydrogels, modeled as Kelvin‐Voigt materials,^[^ ^36^ ^]^ support both dispersive CW turbulence and non‐dispersive Rayleigh wave turbulence (DARWT), influenced respectively by capillary forces and bulk rigidity. To detect both types of surface waves on the same agarose hydrogel, we selected an appropriate agarose concentration (see Figures S1,S2, Supporting Information). This allowed us to explore both capillary and DARWT regimes by varying the excitation frequency ω_0_ (see Figure 1A). The phase velocities vs wave frequency *c*(ω) ≡ ω/*k* were detected for a hydrogel with a reference rigidity where CWs and RWs can coexist (G* ≈ 80 *Pa*), close to the crossover frequency (ω* ≈ 100 *Hz*) (see Figure 1B). The measured wave phase velocities corresponded well with the respective dispersion laws given in Equations (4), (5) (see Figure 1B; Figure S5, Supporting Information). At low frequencies (ω < ω*), the measured phase velocity is nearly constant indicating the presence of RWs, where wave propagation depends solely on bulk rigidity ($c_{RW}^{*}\approx {\left(G^{*}/\rho \right)}^{1/2}\approx 0.35\;m/s$). Higher values of the shear modulus result in higher RW phase velocities (see Figure S5, Supporting Information). At higher frequencies (ω > ω*), the phase velocity starts to rise with increasing excitation frequency indicating the onset of the capillary regime (at ω* ≈ 100 *Hz*) (see Figure 1B). The transition between CW/RW regimes is achieved by changing excitation frequency, though there is a range of frequencies where both wave types do coexist.^[^ ^37^, ^38^ ^]^ After identifying both propagation regimes (CW/RW), we measured the wavefield amplitudes (*A*) to determine the KZ‐spectrum of turbulence as a discrete sequence of nonlinear harmonics visible in the frequency domain within the inertial interval (ω_0_ < ω_*k* + 1_ = *k*ω_0_ < ω_*max*_). We excited the free surface of agarose hydrogel above its linear wavefield response (*A* > *A_NL_* ≈ 0.1 *mm*). Below this threshold (*A* < *A_NL_*) the surface response is characterized by the only appearance of the fundamental mode (ω_0_) (data not shown). The nonlinear surface wave dynamics were tracked using LDV and spectral analysis (see Experimental Section).

Figure 1C (*I*_ω_ against ω_*k*_) shows the KZ‐spectra respectively for high excitation frequency (ω_0_ > ω*), where pure CWs dominate (upper panel), and low excitation frequency (ω_0_ < ω*), where pure RWs dominate (lower panel). The discrete CW‐spectrum follows the scaling law ${I_{\omega }|}_{CW}∼\omega^{-17/6}$ (at ω_0_ > ω*), as predicted in Equation (10).^[^ ^18^, ^19^, ^20^, ^21^, ^22^, ^23^, ^45^, ^46^, ^47^, ^48^, ^49^ ^]^ For the DARWT spectrum (at ω_0_ < ω*), we observe however the scaling law ${I_{\omega }|}_{RW}∼\omega^{-5/2}$, as predicted in Equation (11). This inertial decay interval is followed by a frictional Kolmogorov regime with the steeper power law ${I_{\omega }|}_{frict}∼\omega^{-9/2}$. In all cases, the discretely organized cascades terminate in a continuous background ${I_{\omega }|}_{back}∼\omega^{-2}$, identified as fully developed chaotic turbulence.^[^ ^8^ ^]^ The nonlinear DARWT response appears evidently discrete as a waving cascade of frictionless harmonics characterized by negligible (or minimal) spectral broadening (i.e., Δ_ω_ ≪ ω_0_). This inertial interval extends from the fundamental mode ω_0_, over the maximum frequency, ω_*max*_, the Kolmogorov upper border where the inertial domain ends (ω_*max*_≅ω_*K*_), and turbulent friction dominates on the continuous background.^[^ ^3^, ^8^, ^9^ ^]^ Such a smooth frictional (Kolmogorov‐like) tail observed with Rayleigh waves is characteristic of a KZ‐spectrum of turbulence under discrete wave excitation,^[^ ^51^, ^52^, ^53^ ^]^ quite differently to the sudden drop observed with nonlinear bending waves in flexible plates.^[^ ^31^, ^32^ ^]^

Further physical insight was gained by measuring the nonlinear wave spectra in both capillary and Rayleigh regimes on the same viscoelastic surface, either by changing the excitation frequency or the bulk rigidity. Figure 1D (spectral slope α, against ω) evidences a sharp transition around a critical frequency (ω* ≈ 100 *Hz* for G* ≈ 100 *Pa*), as described in Equation (10), consistent with the phase diagram (Figure 1A), and the phase velocities (Figure 1B). The changes in KZ‐spectral slopes are evident for agarose hydrogels with three different values of bulk rigidity (in the frequency excitation range ω_0_≅20 − 200 *Hz*) (Figure S6, Supporting Information). With very high agarose concentration (*G* ≈ 1.5 *kPa*; *pure DARWT regime*) the slope follows the predicted law in Equation (11), indicating RW presence across the entire excitation range. With medium agarose concentration (*G* ≈ 80 *Pa*; crossover from RW to CWs), two different slopes are measured; for DARWT, α_*RW*_ = 2.46 ± 0.12 ≈ 5/2 at lower subcritical frequencies (ω < ω* ≈ 100 *Hz*); for discrete capillary turbulence, α_*CW*_ = 2.9 ± 0.2 ≈ 17/6 at higher supercritical frequencies (ω > ω*). With low agarose concentration (*G* ≈ 6 *Pa*; *pure CW regime*), surface tension largely dominates over the low bulk rigidity of the highly swollen gel, resulting in a KZ‐spectrum with a consistent capillary slope α_*CW*_ = 2.85 ± 0.12 ≈ 17/6, across all supercritical excitation frequencies, as predicted by the scaling law in Equation (10). By using hydrogels with varying concentrations (and thus different bulk rigidities), at the same excitation frequency, we can also observe the transition from the capillary regime to the DARWT regime (see Figure S6, Supporting Information). In summary, using agarose soft gels, we successfully detected both nonlinear RWs and CWs, each displaying their characteristic KZ‐spectrum. As a rule of thumb, elastic DARWT was detected both by varying the hydrogel's bulk rigidity at a fixed excitation frequency and by changing the frequency at a constant bulk rigidity. The transition between canonical capillary turbulence and elastic DARWT regimes aligns successfully with the HPP theory.^[^ ^36^ ^]^

### 4.2 Injected Energy and Inertial Interval

**Figure 2:**
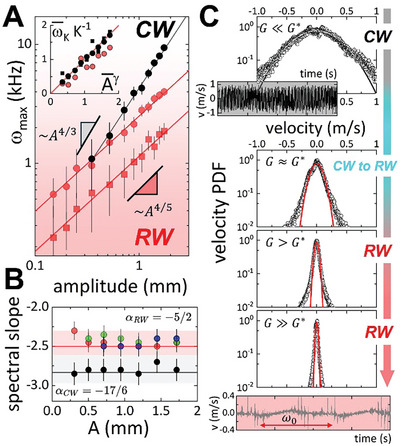
The range for inertial interval [ω_0_ − ω_*K*_] in agarose hydrogels. A) Maximum Kolmogorov frequency (ω_*K*_) at dependence on wavefield amplitude (*A*), as related to power rate injection ($\varepsilon \propto A^{2}\omega_{0}^{2}$). The double‐log plot illustrates scaling $\omega_{max\;}≅\omega_{K}\propto KA^{\gamma }\omega_{0}^{\delta }$ for agarose hydrogels at a constant excitation frequency (ω_0_ = 27*Hz*): crossover RW/CW regime (*G* = 100 *Pa* ≈ G*; black dots); soft‐solid RWs (*G* = 1.0 *kPa*; red dots); rigid solid RW regime (*G* = 1.5 *kPa*; red squares). Best fits align with scaling laws; $\omega_{max}^{\left(RW\right)}\propto K_{RW}A^{4/5}$ for RWs (Equation 15; red lines); $\omega_{max}^{\left(CW\right)}\propto K_{CW}A^{4/3}$ for CWs (Equation 15; black line). The specific pre‐factors, *K_RW_* and *K_CW_*, were obtained as fitting parameters. *Inset)* Master plot frequency‐amplitude showing rescaled inertial interval characteristics ${\bar{\omega }}_{K\;}\equiv \omega_{K}/\omega_{0\;}$ vs $\bar{A}\equiv {\left(A/A_{0}\right)}^{\gamma }$ (under renormalization $A_{0}^{-1}=K^{1/\gamma }\omega_{0}^{(\delta -1)/\gamma }$). B) Invariance of KZ‐spectral slopes with the amplitude of excitation *A* for different shear rigidity: *RW regime G* > G*) Solidlike hydrogels with *G* = 0.5 *kPa* (red dots); *G* = 1.0 *kPa* (blue dots); *G* = 1.5 *kPa* (green dots); *CW regime G* < G*); liquidlike hydrogels with *G* = 6*Pa* (black dots). All data have been obtained at the equalized excitation frequency ω_0_ = 48 *Hz*. C) Probability density function (PDF) for the dimensionless normal surface velocity *v*/〈*v* ^2^〉^1/2^ with increasing rigidity across propagation regimes (at fixed ω_0_ = 48 *Hz*); upper panel: pure CW waves for *G* = 6 *Pa*; lower panels (from top to bottom): pure RWs for *G* = 0.5 *kPa* (top), *G* = 1.0 *kPa* (middle) and *G* = 1.5 *Pa* (lower). The straight lines represent the best fit to the normal Gaussian distribution. *Insets)* Time‐series for surface velocities showing chaotic fluctuations for liquidlike capillary turbulence (upper; grey background), and low amplitude fluctuations for solidlike Rayleigh waves superposed on the carrier fundamental frequency (ω_0_ = 48 *Hz*) (bottom; reddish background). Note the gradual decrease of long PDF tails corresponding to chaotic displacements as the system transitions from the liquid‐like, dispersive CW regime, down to the solid‐like, dispersionless RW regime.

**Figure** **2** presents the main results referring to the size of the inertial interval [ω_0_ → ω_*max*_] dependent on the injected energy density ($\varepsilon \equiv A^{2}\omega_{0}^{2}$). Key DARWT features differentiate dispersionless RWs from genuinely dispersive CWs (see Section 3). Figure 2A (double log plot for ω_*max*_ against *A*) illustrates the distinct scaling behavior of the Kolmogorov frequency for DARWT compared to CW turbulence as the wavefield excitation amplitude increases. Specifically, a larger inertial interval was observed with increasing *A* (Figure 2A), and higher excitation frequency ω_0_ (Figure S7, Supporting Information). According to dimensional analysis within the ZF‐theory, the maximum Kolmogorov limit, $\omega_{max}≅\omega_{K}\left(A,\omega_{0}\right)\propto KA^{\gamma }\omega_{0}^{\delta }$, follows the power‐law derived for RWs (γ_*RW*_ = 4/5; δ_*RW*_ = 7/5; Equation 14), or the well‐established formula for CWs (γ_*CW*_ = 4/3; δ_*RW*_ = 23/9; Equation 15). Both scaling laws were tested using agarose hydrogels with varying rigidities near the critical RW/CW crossover (at G* ≈ 80 − 100 *Pa*), by generating nonlinear surface wavefields across a broad excitation range ($\omega_{0}\approx 20-200Hz≷\omega^{*}\approx 100\;Hz$). For very soft hydrogels (*G* ≈ 6 *Pa* ≪ G*), we observed pure CW behavior (γ = 1.34 ± 0.04 ≈ γ_*CW*_), aligning perfectly with the ZF‐ power law in Equation (15).^[^ ^8^, ^46^ ^]^ Conversely, for the stiffest hydrogels considered (*G* ≈ 1 − 1.5 *kPa* ≫ G*), elastic DARWT appear throughout the entire excitation range (γ = 0.78 ± 0.05 ≈ γ_*RW*_), effectively decreasing the inertial interval toward lower Kolmogorov frequencies with increasing concentration. Generally, the scaling laws for the inertial interval size incorporate friction‐inertia amplitudes *K*(ρ, η), which are proportional to bulk density (ρ), but also inversely proportional to viscosity (η), hence determining the damping rate of the nonlinear harmonics. Increasing agarose hydrogel concentration in our experiments raises both rigidity (*G*), and bulk viscosity (η) (see Note S1, Figures S1,S2, Supporting Information), leading to effectively shorter inertial interval (Figure 2A; Figure S7, Supporting Information). Rescaling Kolmogorov frequencies ${\bar{\omega }}_{K}\equiv \omega_{K}/\omega_{0}$, against renormalized amplitudes $\bar{A}\equiv A/A_{0}$ (provided $A_{0}^{-1}=K^{1/\gamma }\omega_{0}^{(\delta -1)/\gamma }$), produces a master plot ${\bar{\omega }}_{K}K^{-1}={\bar{A}}^{\gamma }$ (Figure 2A; inset), demonstrating the universal DARWT‐class across propagation regimes and material characteristics. Furthermore, we assess potential amplitude‐dependent artifacts within the characteristic dynamic scaling reflected in the KZ spectra. Figure 2B displays the constant spectral slope observed against increasing *A*, for both DARWT and capillary turbulence, indicating no significant change within the considered amplitude range. In these experiments, wavefield excitations were conducted within frequency ranges where typical wavelengths are millimeters. Hence, in the large 15 cm diameter vessel used under deep water conditions, we can consider surface nonlinear wave dynamics free from wall boundary/bottom influences. This instrumental issue is crucial, as wall influences can affect important wave turbulence spectra, unlike in laboratory flumes with gravity wave turbulence.^[^ ^1^, ^3^ ^]^ With transverse excitation amplitudes ranging from 1–4 mm as a maximum, the nonlinear waves remain within the weak wave turbulence regime, ensuring λ ≪ *D* and *u*≅*v*/ω ≪ *h*, hence avoiding for any boundary influence with a spurious effect on wave discretization. Likely, the experimental results in Figure 2B confirm no artifacts in the characteristic scaling observed under the broad range of experimental conditions considered.

### 4.3 Velocity Distributions

Despite the discreteness of the harmonic cascades, the nonlinear modes emerged over a continuous background indicative of underlying chaotic turbulence, $I_{back}∼\omega^{-2}$ (Figure 1C),^[^ ^8^ ^]^ as previously demonstrated in turbulent systems under bulky excitations [¡Error! Marcador no definido].^[^ ^54^ ^]^ To investigate this point with surface turbulence, we built the probability density function (PDF) of the transverse wavefield velocities (*v*), using dynamic data from LDV (see Experimental Section). Figure 2C shows the experimental PDFs during the dynamic crossover from surface liquidlike capillary turbulence on the softest hydrogel (top panel), down to bulky elastic solidlike DARWT on stiff hydrogels (lower panels). The nonlinear CW manifold exhibits a broad, near‐Gaussian PDF corresponding to the Maxwell‐Boltzmann velocity distribution of a disordered liquid‐like fluctuating ensemble as revealed in the velocity time series (Figure 2C; upper inset). However, the RW regime shows a progressively narrower, Debye‐like, distribution typical of more ordered dynamic states. Notably, the stochastic velocity distributions focus as the concentration increases, with higher rigidity resulting in more ordered surface DARWT wavefields. The observed decreasing velocity variances align with narrower spectral linewidths decreasing Arrhenius‐like with rigidity, Δ_*CW*_∝*exp*(− *G*/G*) (Figure S8, Supporting Information), highlighting the exactness of nonlinear couplings at connection with the interaction lifetimes controlled by kinetic energy ($\Omega \equiv \tau_{NL}^{-1}\propto E_{k}$). For dispersionless acoustic RWs, discrete resonances occur with spectral line narrowing characteristic of solidlike behavior (Δ_*RW*_ → 0 for *G* ≫ G*), while CWs exhibit broader linewidths due to liquidlike dispersivity (Δ_*CW*_ ≫ 0 for *G* → 0) (Figure S8B, Supporting Information). Arguably, dispersive CW couplings are more affected by multiple scattering (Ω_*CW*_ ≥ Δ_*CW*_), than dispersionless acoustic couplings in RWs (Ω_*RW*_ ≈ Δ_*RW*_ ≈ 0), enabling conservative DARWT under exact resonances (Ω_*RW*_ → 0) (Note S3, Supporting Information). The surface DARWT wavefield remains so well‐focused over the mostly stochastic background that the fundamental resonance is still detectable modulating the stochastic time series (see Figure 2C; bottom inset).

### 4.4 Three‐Wave Coupling Interaction

**Figure 3:**
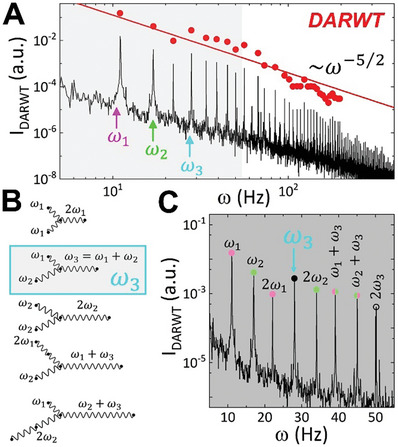
Three‐wave interaction in nonlinear Rayleigh waves under sum‐frequency resonance. A) DARWT spectrum obtained under *dichromatic primed excitation* applied over a rigid hydrogel supporting Rayleigh waves (*G*  =  1.5 *kPa* ≫ G*). The excitation frequencies are chosen to be prime numbers (ω_1_ = 11 *Hz*,   ω_2_ =  17*Hz*). The DARWT response is identified as the sum‐frequency second harmonic, ω_3_ = ω_1_ + ω_2_ = 28*Hz*, and their respective higher harmonics in a nonlinear energy cascade of dispersionless Rayleigh modes. The symbols depict spectral peak amplitudes, and the straight line corresponds to the RW‐scaling law *I*_ω_∝ω^−5/2^. B) Triadic coupling interactions as exact three‐waves resonances. The expected wave couplings are depicted as triadic interaction diagrams from the primed frequencies involved (ω_1_ and ω_2_). A sum‐frequency parent is expected as ω_3_ = ω_1_ + ω_2_ = (outlined in cyan). C) Second‐harmonic generation region close to the fundamental excitation modes (a grey‐shadowed spectral region in A). At the very beginning of the harmonic cascades, we found the fundamental modes (ω_1_ and ω_2_), their first harmonics (2ω_1_ and 2ω_2_), and their second‐harmonic pure sum‐frequency superposition (ω_3_ = ω_1_ + ω_2_; marbled in cyan). Subsequent harmonics show higher‐order combinations of the parent modes (2ω_1_ +  ω_2_ = ω_1_ + ω_3_, 2ω_2_ +  ω_1_ = ω_2_ + ω_3_, etc.).

**Figure** **3** illustrates how nonlinear Rayleigh waves (RWs) interact through exact three‐wave resonant coupling spanning over several scales of the inertial interval. From the kinetically posed ZF‐theory, given the dispersion relation of the surface waves, either CWs or RWs, we can predict the number of interacting waves that lead to an effective energy cascade from large to small scales. This nonlinear wave interaction in kinetic wave turbulence is determined by the spatial structure of the interacting modes as imposed by the dispersion relation, suggesting that non‐dispersive DARWT primarily involves exact three‐wave interactions as the main mechanism for energy cascade in a discrete KZ‐like spectrum. To demonstrate this energy principle, we conducted a combinatorial sum‐frequency experiment with dichromatic excitation, using two distinct frequencies tagged as prime‐numbers (ω_1_ = 7*Hz* and ω_2_ = 11*Hz*), exciting DARWT in a quite rigid agarose hydrogel (*G* ≈ 1 *kPa* ≫ G*). The high shear rigidity ensures both excitation frequencies lie within the RW regime (see Figure 1A). As a proof‐of‐concept, prime frequency combinatory allows us to evaluate nonlinear interactions by analyzing independent spectral peaks corresponding to clustered “wave sisters” and their respective primed harmonics.^[^ ^11^, ^19^ ^]^ As shown in Figure 3A, the KZ‐spectrum obtained by dichromatic excitation follows the same RW turbulence scaling law, $I_{\omega }{|}_{RW}^{3D}\;∼\;\omega^{-5/2}$, as also predicted for monochromatic excitation (Equation (14)). Figure 3B illustrates the potential triads involved in exact three‐wave resonant interactions. As a rule of thumb, Figure 3C examines the sum‐frequency harmonics detected near the fundamental excitation frequencies (ω_1_ and ω_2_), both engendering triads under primality as constituted by the three minimal “sisters”, ω_1_, ω_2_ and ω_3_ = ω_1_ + ω_2_ (none of them a multiple of each other). These results confirmed the three‐wave genesis of the DARWT energy cascade, with discrete peaks corresponding to the fundamental frequencies (ω_1_ = 7 *Hz* and ω_2_ = 11 *Hz*), and their lowest harmonics (2ω_1_ = 14*Hz* and 2ω_2_ = 22*Hz*), as well as the combined sum‐frequency first triadic sister mode (ω_3_ = ω_1_ + ω_2_ = 18*Hz*). Deliberately, we selected triadic excitation with prime frequencies to avoid spurious recombination into quartet modes.^[^ ^19^ ^]^ For non‐prime composite frequencies (e.g., ω_1_ = *n*ω_0_ and ω_2_ = *m*ω_0_), exact interactions can form either a triad, ω_3_ = ω_1_ + ω_2_ = (*n* + *m*) ω_0_, or quartets ω_3_ + ω_4_ = ω_1_ + ω_2_, involving ascendant harmonics ω_4_ = (*m* + *k*)ω_0_ > ω_2_ and descendant subharmonics ω_3_ = (*n* − *k*)ω_0_ < ω_1_, both dismuted under inelastic scattering.^[^ ^19^ ^]^ While sister triads are clear from the generating primed parent waves (ω_1_ and ω_2_), dismutation quartets are hard to distinguish from higher harmonics of the common parental frequency (ω_0_), except for the absent descendant subharmonics. In our frequency‐primed experiment, three‐wave nonlinear resonance always involves higher (non‐dismuted) harmonics of the two primary frequencies, such as second harmonics, 2ω_1_ + ω_2_ = ω_1_ + ω_3_, ω_1_ + 2ω_2_ = ω_2_ + ω_3_, third ones ω_1_ + 3ω_2_ = 2ω_2_ + ω_3_, 2ω_1_ + 2ω_2_ = 2ω_3_, 3ω_1_ + ω_2_ = 2ω_1_ + ω_3_, and so on. As the inertial range extends to higher frequencies, the KZ‐spectrum becomes increasingly intricate, with more combinations of higher harmonics independently emerging under parental primality from the elemental sister triad (ω_1_, ω_2_ and ω_3_) (see Figure 3B; Figure S9, Supporting Information).

### 4.5 Universality Features: DARWT Validation

**Figure 4:**
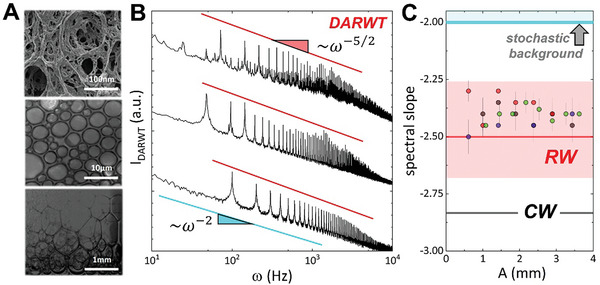
Universality features of RW turbulence. A) Structural characteristics of different viscoelastic materials used in our experiments (from top to bottom): polyacrylamide hydrogel with nanoscopic porosity (imaged by SEM); oil‐in‐water emulsion stabilized as a soft solid with aescin surfactant (under an optical microscope); heterogenous solidlike dry foam stabilized with aescin (Photography with CCD camera). Note large differences in length scales. B) KZ‐DARWT spectra for the above viscoelastic materials excited nonlinear within their respective RW regimes (ω_0_ < ω*): polyacrylamide hydrogel (ω_0_ = 70 *Hz*); liquid emulsion (ω_0_ = 50 *Hz*); solidlike foam (ω_0_ = 100 *Hz*). The red lines with their corresponding circles follow the power law RW‐dependence *I*_ω_∝ω^−5/2^. The spectra are vertically shifted for clarity. DARWT spectra consistently form on a Lorentzian‐like background, *I_back_* ∝ω^−2^, compatible with fully developed stochastic turbulence (Landau turbulence). C) Invariance of the spectral slopes with excitation amplitude of the wavefield, at the same frequencies as represented in panel B), for polyacrylamide hydrogel (red dots), agarose (blue dots), w/o emulsion (green dots) and solidlike foam (black dots). The straight lines indicate theoretical scaling exponents: bottom‐up, α_*CW*_ = −17/6 (capillary turbulence; black); α_*RW*_ = −5/2 (DARWT; red); α_*Landau*_ = −2 (stochastic turbulence; cyan). The reddish dashed region marks the variability range for the DARWT scaling exponent from Figure 2B, while the bluish region represents the domain of developed stochastic turbulence in Landau's continuum; see main text for details.

Ultimately, **Figure** **4** demonstrates that nonlinear Rayleigh wave dispersionless propagation in soft solids is a universal characteristic leading to elastic DARWT in different classes of soft solids. We have already evaluated the theoretical premises of statistical kinetic DARWT turbulence applied to RWs on the surface of agarose hydrogels and here aimed to show the universality of the predicted DARWT spectrum using various viscoelastic materials with a soft solid character. The goal is to demonstrate the presence of nonlinear RWs in soft solids with different non‐Newtonian flow properties. Figure 4A displays several examples of viscoelastic materials used in this work: polyacrylamide (PA) hydrogel, liquid o/w‐emulsion, and solidlike foam. The latter was formed by shaking β‐aescin with water, creating a very stable solid foamy structure due to the extraordinary strengthening properties of aescin as a surface shear stiffener.^[^ ^41^ ^]^ These materials exhibit vastly different characteristic scales, ranging from 100 nm for the tightly crosslinked PA hydrogel, to 10 µm for the diluted o/w‐emulsion and 1 *mm* for the solid‐like aqueous foam (Note S2, Supporting Information). Detailed rheological studies show that elastic behavior dominates over viscous damping in all cases (Figure S3, Supporting Information). Thus, at low frequencies of excitation and high amplitudes, the RW spectrum of turbulence should be observed. The HPP theory predicts such surface waves for a Kelvin–Voigt model material when η < *G*/ω, meaning bulk rigidity dominates over viscosity in all these soft solid materials behaving predominantly elastic (Figure S3, Supporting Information).

After determining the corresponding DARWT regime for each soft solid material based on their rheological measurements (see Figures S1–S3, Supporting Information), their KZ‐spectra of kinetic elastic turbulence was determined in all cases. Figure 4B displays the characteristic fingerprint of nonlinear RWs in agreement with the scaling law in Equation (11). All materials show the same class of DARWT spectrum as the agarose hydrogel above discussed. Figure 4B shows discrete modes emerging as exact three‐waves resonances (Ω_*N* = 3_ ≪ Δ_ω_) under DARWT‐predicted spectral envelope, *I_DARWT_* ∝ω^−5/2^ (Equation 14). These spectra overlay a continuous Lorentzian‐like background *I_back_* ∝ω^−2^, characteristic of fully developed fluid turbulence, likely dominated by chaotic Landau dynamics across the several scales probed.^[^ ^8^ ^]^ Even for dynamically heterogeneous foams, the same behavior is observed regarding the terminal Kolmogorov interval of limiting dissipative frequencies, at ω ≥ ω_*K*_ (Figure S10, Supporting Information). Validation of the DARWT spectral distribution is further assessed by measuring the consistency of the spectral slopes as the wave field amplitude *A* is changed, as depicted in Figure 4C. Indeed, there was no appreciable variation over a wide range of excitation amplitudes, and the observed spectral dependence remains scale‐invariant, $I_{\omega }{|}_{RW}^{3D}\;\propto \omega^{-5/2}$, marking the universal fingerprint of the elastic Rayleigh wave spectrum of turbulence.

## 5 Discussion

Our study investigates nonlinear Rayleigh waves (RWs) kinetically excited on viscoelastic materials, including hydrogels, emulsions, and foams. By excluding finite‐size effects beyond monochromatic RW discretization, we focused on the inertial interval as detected inviscid in the KZ‐spectrum, highlighting resonant weak coupling on the free surface of these soft solid materials. When bulk rigidity or surface tension dominated viscous dissipation, nonlinear, nondispersive RW/CWs emerged as discrete harmonics (ω_*k*_ = *k*ω_0_ for *k* ≥ 2), with a very characteristic power‐law envelope $I_{\omega }{|}_{RW}^{CW}\propto \omega_{k}^{-\alpha }$, akin to the KZ‐spectrum of kinetic turbulence.^[^ ^23^ ^]^ Relevantly, the power‐law decay dependence along discrete harmonics reflects surface motion kinetic excitation resonating with the fundamental driving mode across scales. Indeed, we conceived the inertial cascades of discrete nonlinear RW/CWs in similar kinetical terms as theoretically described by Zakharov and Filonenko (ZF) for statistical weak turbulence.^[^ ^8^ ^]^ Based on ZF‐theory through dimensional analysis, we predicted, and experimentally confirmed, the corresponding KZ‐spectrum of discrete acoustic Rayleigh wave turbulence (named DARWT), which shows a characteristic direct energy cascade like the continuum spectrum described by the statistical ZF‐framework.^[^ ^1^, ^23^ ^]^ DARWT was observed under enough shear rigidity, exhibiting key features of kinetically conservative elastic turbulence as observed in viscoelastic polymers under nonlinear flow.^[^ ^13^ ^]^ Hence, we have analyzed DARWT in two differentiated empirical settings. First, we determined the surface wave propagation regime at the dependence of liquid‐like capillary or solid‐like Rayleigh behavior, identifying whether surface tension or bulk rigidity respectively governs the nonlinear wave turbulent dynamics, thus leading in each respective case to either discrete capillary turbulence or elastic DARWT as based in surface Rayleigh waves.^[^ ^35^ ^]^ Second, we analyzed the discrete KZ‐spectra for both wave types and examined the transition from liquidlike capillary to elastic solidlike DARWT by appropriately varying either, the frequency of excitation, or the bulk shear rigidity dominating over friction. The observed changes in the nonlinear CW/RW regimes aligned perfectly with validating predictions from the classical HPP theory for Rayleigh wave propagation on viscoelastic materials.^[^ ^36^ ^]^

For the first time, we have revealed the existence of the new DARWT universality class, as pure‐elastic (dispersionless) discrete wave turbulence based on nonlinear Rayleigh waves excited under monochromatic excitation. We have considered very different viscoelastic materials with dissimilar structural characteristics. For DARWT, we have experimentally demonstrated the kinetic scaling law that describes the turbulent inertial interval *I*_ω_|_*RW*_ ∝ω^−5/2^ (under elastic scaling α = 5/2). This spectral fingerprint corresponds to the universality class for dispersionless acoustic waves (ω ≈ *ck*), under exact three‐wave resonance (*N* = 3), in 3D (*d* = 3), as theoretically predicted from the kinetic ZF‐framework.^[^ ^1^, ^23^ ^]^ Based on sum‐frequency experiments using two prime exciting waves ω_1_(*k*_1_) and ω_2_(*k*_2_), both overlapping temporally and spatially on the soft solid surface as collinear modes, we revealed the exact nature of three‐wave interactions for non‐dispersive RWs, likewise dispersive CWs, leading to near conservative coupling into sister triads i.e., ω_1_(*k*_1_) + ω_2_(*k*_2_) = ω_3_(*k*_3_) and *k*_1_ + *k*_2_ = *k*_3_, stablished under minimal resonance broadening Δ_ω_≅0. These weakly nonlinear couplings preserve resonant three‐waves quasi‐exactness on both, the discrete solidlike DARWT (Ω_*N* = 3_ ≪ Δ_*RW*_ → 0), and liquidlike capillary dispersive cascades (Ω_*N* = 3_ ≈ Δ_*CW*_ > 0), which appear inertially superposed on a continuous background characteristic of Kolmogorov‐Landau chaotic turbulence.^[^ ^8^ ^]^ Specifically for elastic DARWT, we showed the inertial KZ‐spectrum increasing with excitation amplitude *I_A_* |_*RW*_ ∝*A* ^−5/2^, under invariant elastic scaling across a wide range of amplitudes and frequencies, hence confirming the validity of the kinetical weak‐nonlinearity approximation across the inertial interval. For the newly demonstrated kinetic DARWT fully developed, the inertial cascade spans practically inviscid from the fundamental mode down to the Kolmogorov scale of frictional dead. Furthermore, we have explored the residual spectral broadening with increasing excitation amplitude in the inertial interval of the corresponding RW/CW cascades. We found that nonlinear RWs propagate dispersionless under minimal frictional losses compared to their dispersive CW counterparts. The inertial peaks were found to be narrowest for nonlinear DARWT modes, while broader dissipative peaks were observed for capillary modes. All these phenomenological properties were successfully predicted from scaling analysis for three‐waves interactions in 3D (*N* = 3,  *d* = 3), as based on the statistical theory formulated by Zakharov and Filonenko,^[^ ^23^ ^]^ under the discreteness provision in the Kartashova framework.^[^ ^11^, ^19^ ^]^ Our proof‐of‐concept study demonstrates that exact three‐wave interactions, particularly through nondispersive Rayleigh waves, dominate conservative energy transfer in acoustic turbulence on soft solids. The validating experiments with agarose gels and other viscoelastic solids have revealed that exact three‐wave resonance sustains discrete Rayleigh wave turbulence (DARWT), underscoring its critical role in stable energy transfer over scales. These results highlight several unprecedented features of weak turbulence present in the nonlinear DARWT cascades excited on agarose hydrogels, particularly, the non‐dispersive KZ‐spectrum (*I_RW_* ∝*A*ω^−5/2^), the invariant decay of the stochastic background (*I_back_* ∝*A*ω^−2^), and the Kolmogorov's frequency ($\omega_{K}\left(\varepsilon \right)\propto A^{4/5}\omega_{0}^{7/5}$). These spectral features indicated a discrete sequence of exactly resonant modes focused on a reduced velocity ensemble up to a maximum Kolmogorov frequency related to the energy excitation. The observed scaling behavior aligns well with our dimensional analysis of the phenomenologically identified RW universality class (see Section 3).

This study highlights the practical applicability of DARWT spectral characterization via laser Doppler vibrometry for understanding nonlinear wave interactions and advancing rheological analysis of soft solids. By linking viscoelastic properties to Rayleigh wave turbulence, our new theoretical framework combined with state‐of‐the‐art experiments enables non‐invasive, contactless mechanical characterization across spatiotemporal scales. The revealed universal KZ‐spectral features of the inertial interval provide insights into energy exchange and material mechanics. Well‐known scaling laws for phase velocity and nonlinear DARWT cascades have been demonstrated to correlate with rheological properties, offering a novel approach to analyze viscous and elastic behavior over a broad frequency range (1–1000 Hz), surpassing traditional rheology limitations. Our experimental findings for rheologically simple Kelvin‐Voigt materials align perfectly with weak kinetic theory, encouraging further theoretical investigation on more complex rheological responses.

## 6 Conclusions

In summary, this study reveals the unique combination of kinetically statistical features from classical ZF‐theory and deterministic aspects of discrete wave turbulence in the observed DARWT. Our findings establish a universal KZ‐spectrum for elastic DARWT in bulk materials, governed by exact three‐wave coupling interactions. The robustness of our theoretical predictions across diverse soft viscoelastic materials demonstrates the universal applicability of this approach, independent of structural or rheological differences. This method offers a promising complementary tool for surface rheological characterization, extending beyond traditional techniques like microscopy and indentation, and enabling analysis of complex materials over a wide frequency range. By examining the universality of DARWT across materials and instruments, we provide a comprehensive framework for exploring wave dynamics and nonlinear interactions that traditional methods cannot address. This study bridges gaps in understanding complex, non‐Newtonian flows in soft viscoelastic materials, offering a theoretical foundation for modeling surface acoustic wave turbulence and energy cascades. Our conclusions open new avenues for experimental, computational, and theoretical research, with potential applications in different viscoelastic settings. This work paves the way for further experimental and theoretical exploration of nonlinear surface wave dynamics mediated by capillary and elastic forces.

## Conflict of Interest

The authors declare no conflict of interest.

## Supporting information



Supporting Information

## Data Availability

The data that support the findings of this study are available in the supplementary material of this article.
