# Supplementary material for: Acoustic Rayleigh Wave Turbulence in Soft Viscoelastic Matter
Source: Adv Sci (Weinh). 2025 Feb 22;12(15):2407528. doi: 10.1002/advs.202407528 (PMC12005792; doi:10.1002/advs.202407528)
Supplement: Supplementary file 1 — Supporting Information [file ADVS-12-2407528-s001.docx]

**Acoustic Rayleigh wave turbulence in soft viscoelastic matter**

Mikheil Kharbedia, Horacio Lopez-Menéndez, Basilio Javier García-Carretero, Manuel G. Velarde and Francisco Monroy.

**SUPPLEMENTARY MATERIALS**

**Supplementary Notes SN1-SN3**

**Supplementary Figures S1-S10**

**Supplementary Note SN1. Rheological model** [1, 2]**.**

As the composite systems we have used in our experiments offer superposed liquid-like viscous and solid-like elastic features we need to account for both. The standard Newtonian fluid under tangential shear deformation follows the Newtonian law of viscosity:

$$\begin{aligned} \tau_{l}=\eta\frac{d\gamma}{dt}, \#SN1 \end{aligned}$$

where $\eta$ and $\gamma$ are the dynamical (shear) viscosity and shear deformation, respectively. Analogously, the standard Hookean solid follows the law:

$$\begin{aligned} \tau_{s}=G'\gamma, \#SN2 \end{aligned}$$

where $G´$ is the elastic modulus of the material.

The oscillation rheology measurement determines the behavior of the material under oscillatory shear deformation. Thus, we can set:

$$\begin{aligned} \tau_{l}=\eta\frac{d\gamma}{dt}= \eta\omega\gamma_{0}\sin\left( \omega t+\phi\right), \#SN3 \end{aligned}$$

$$\begin{aligned} \tau_{s}=G'\gamma= G'\gamma_{0}\sin\left( \omega t \right), \#SN4 \end{aligned}$$

where $\phi$ is the phase difference between liquid-like and solid-like responses.

It seems useful to define a complex modulus $G^{*}$:

$$\begin{aligned} G^{*}=G^{'}+iG^{''}. \#SN5 \end{aligned}$$

With real part:

$$\begin{aligned} G^{'}=G^{*}cos\phi=\frac{\tau_{0}}{\gamma_{0}}cos\phi, \#SN6 \end{aligned}$$

and imaginary component:

$\begin{aligned} G^{''}=G^{*}sin\phi=\frac{\tau_{0}}{\gamma_{0}}sin\phi. \#SN7 \end{aligned}$

The first term $G$ is known as *storage* modulus and accounts for the elastic behavior of the material. $G''$ is called *loss* modulus and estimates the viscous behavior of the material. Then it seems natural that to describe the dynamical response of the material we introduce a *complex* viscosity, *η**, so that:

$$\begin{aligned} \eta^{*}=\eta^{'}+i\eta^{''}=\frac{G^{*}}{i\omega}. \#SN8 \end{aligned}$$

From a phenomenological point of view $G$ describes the elastic response of the material while $\eta$ is its frequency dependent viscosity. Such a superposition of viscous and elastic responses of a material is what the Kelvin-Voigt model of a viscoelastic model material provides with the frequency dependent viscosity [2]:

$$\begin{aligned} \eta\left( \omega\right)=\eta^{'}+\frac{G}{i\omega}. \#SN9 \end{aligned}$$

In our case the viscous response can be attributed to the water in hydrogel medium whereas the elastic response is due to the homogenous 3D polymeric structure [2].

Noticeably in Eq. *SN9* is that $G$ is introduced independent of shear frequency. In accordance with the above description, we have performed an oscillatory amplitude sweep measurement (constant frequency while changing the amplitude of deformation). In this way, we can determine the regime of deformation where linear viscoelasticity occurs as a Kelvin solid. To assess the dynamical response, we can arbitrarily choose a value of linear deformation within the linear regime and vary the shear frequency at constant strain. Dividing the shear modulus ($G$) by the oscillation frequency ($\omega$), one can estimate the actual value of the bulk frictional resistance due to solidlike rigidness.

**Supplementary References**

[1] G. Schramm, *A Practical Approach to Rheology and Rheometry*, pp. 20-25 (Haake, Karlsruhe,1994).

[2] J. L. Harden, H. Pleiner, P. A. Pincus. J. Chem. Phys. **94**, 5208 (1991).

**Supplementary Note N2. Viscoelastic materials used as soft solids: structural characterization by optical microscopy and SEM.**

**Table S1. Viscoelastic materials.** The table below lists all viscoelastic materials classified as soft solids used in this work. The first column details the structurally distinct materials tested (physically entangled agarose gels, chemically crosslinked polyacrylamide hydrogels, both solid but highly swollen, Escin-stabilized dry foam as a mechanically resilient soft solid, and mineral oil-in-water emulsion as a paradigm of flowing soft viscoelastic structure). Subsequent columns indicate concentration ($c$), bulk rigidity ($G$), dynamic viscosity ($\eta$) and frequency range ($\omega$), relevant to CW and RW turbulence studies with these materials. Solid concentrations are specified as millimolar, % by mass (% w/w), or % by volume (% v/v). For materials studied systematically (e.g., soft agarose gels and solidlike foams), full concentration ranges and corresponding structural properties are provided, with further details in Supplementary Figure S1, and in Figure SN3-2b of Supplementary Note SN3.

**
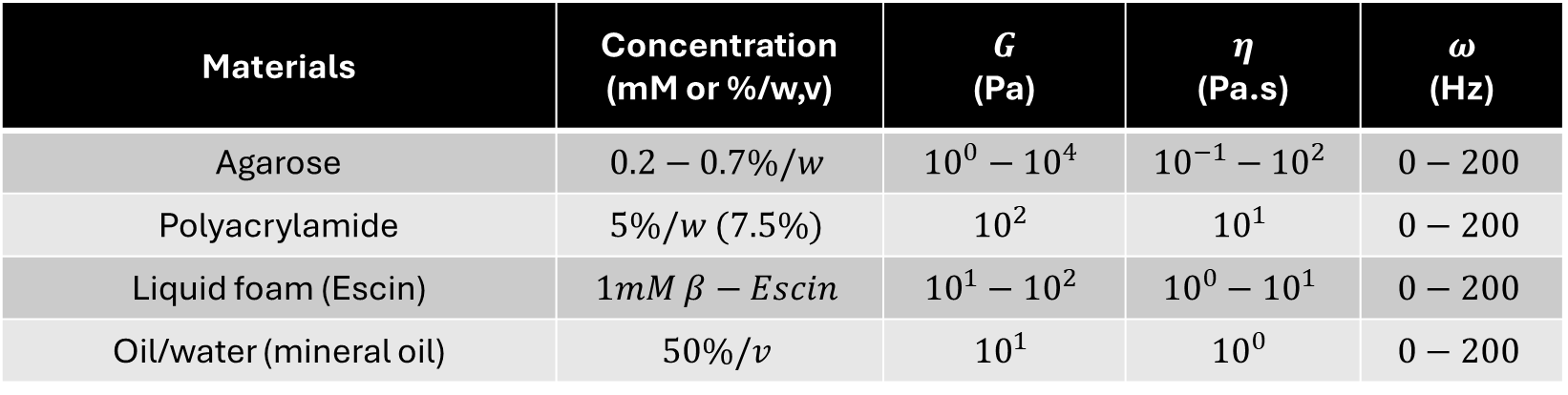
**


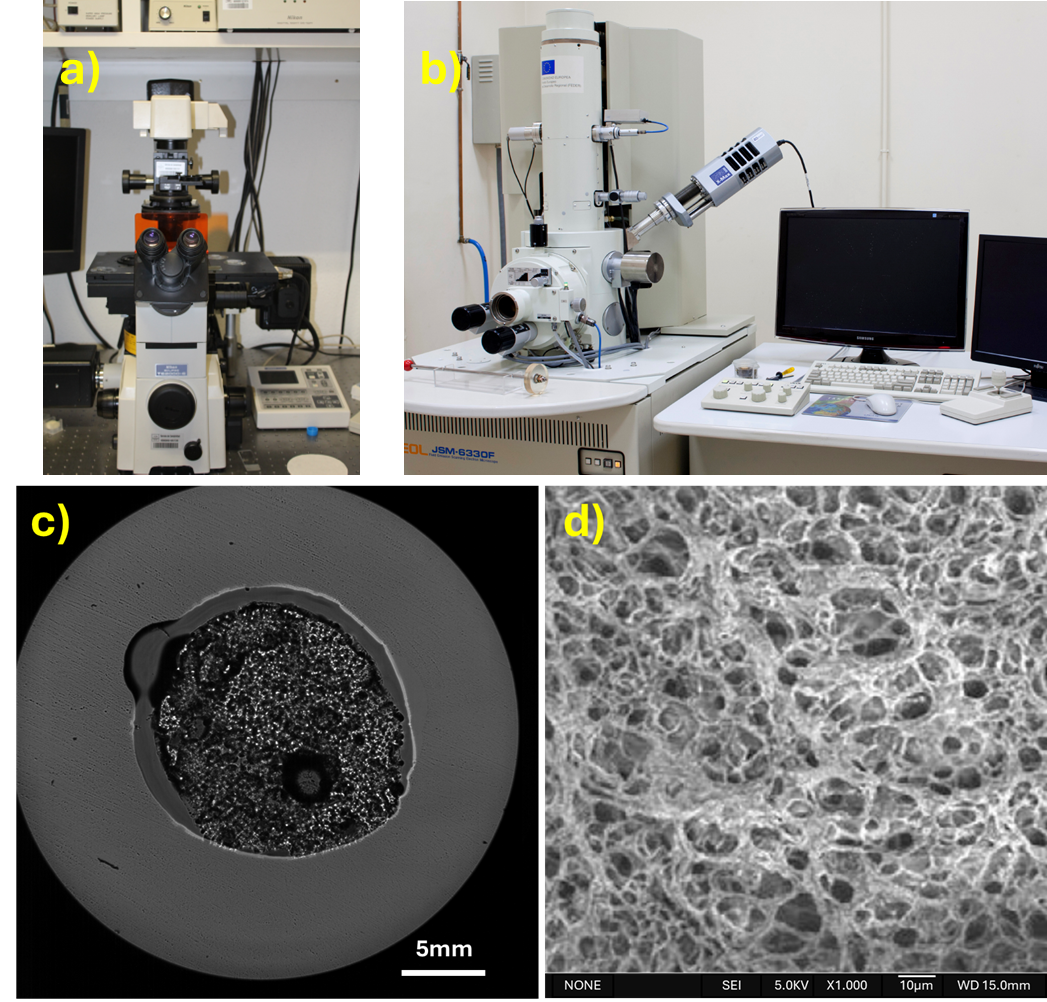


**Figure SN2-1. Experimental setting for structural characterization. a) Optical microscope** for granulometry and cell size characterization in the microscale (Nikon TE2000, bright field mode with a high luminosity objective; x100, NA 1.45,). **b) Scanning electron microscopy (SEM)** for mesoscopic structural characterization of material porosity (JEOL JSM-6330F). **c) Typical heterogenous aspect** of soft solid materials as observed under the optical microscope. This microdroplet sample corresponds to polyacrylamide hydrogel synthetized with 5% in mass of polyacrylamide and 7.5% in mass of bis-acrylamide (crosslinker), mixed in distilled water (milliQ). **d) SEM characterization of swollen polyacrylamide mesoporous gels with a high porosity.**  Before the polymerization is complete, a drop is placed on a silica wafer and sampled and sampled with SEM at 5kV, x1.000 magnification and at 15mm of working distance. Note the highly homogeneous porosity of these gels in the microscale (typical pores size 5 μm). These structural conditions are maintained for those hydrogels used to study surface RW turbulence spectra.


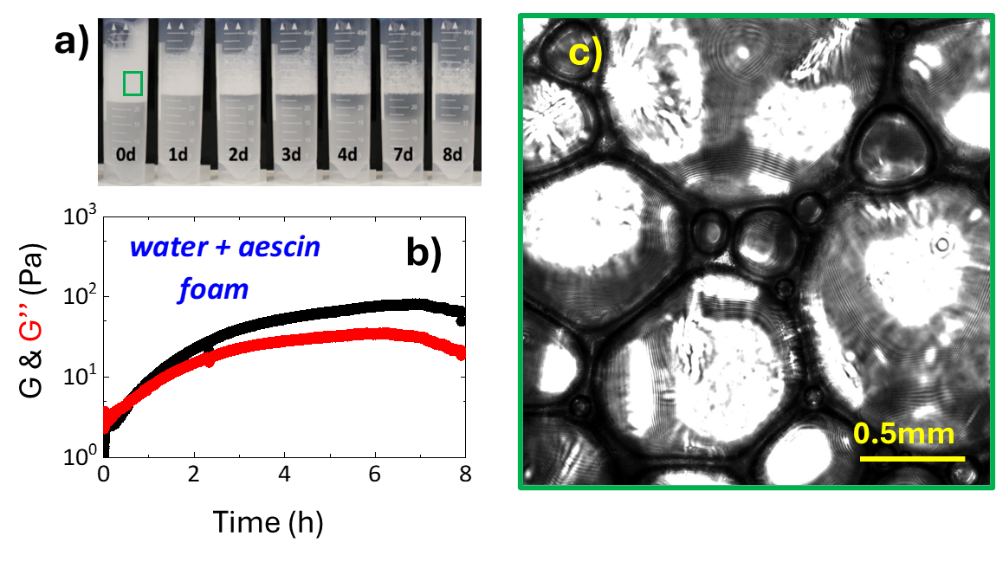


**Figure SN2-2. Escin-stabilized dry foams: Rheological and optical characterization. a) Soft-solid foam generated by shaking** water and Escin at 1mM (near the critical micelle concentration). The stability of the foam is shown through images over several days, indicating a highly viscoelastic structure lasting up to 8 days. **b) Rheological assessment** confirms sufficient drainage for predominantly elastic behavior. Oscillatory rheology under cone-plate geometry (~ $500\mu m$ gap, $\gamma=1 \%$ constant shear amplitude, $\omega=1Hz$ constant shear frequency; see Figure SN1-1 for a detailed explanation). Rheology tests show that transient viscoelasticity evolves as a rheopectic solid. Initially, liquid viscosity dominates, but drainage and network strengthening lead to a predominantly elastic foam with a storage modulus ($G$) greater than the lost modulus ($G''$). After a few hours, the foam reaches a plateau, suitable for LDV measurements, which are completed within 10 minutes to avoid rheological drift. At plateau, bulk rigidity is$G_{max}\approx10Pa$ and shear viscosity $\eta={G''}/\omega\approx3Pa.s$. **c) Structural mesoscopic characterization**. Optical microscopy of the foam after a few hours of curation reveals heterogeneous plateau cells with diameters of 0.5–1 mm (50x magnification, scale bar = 0.5 mm).

**Supplementary Note N3. Nonlinear interaction rate calculated in terms of spectral broadening of vessel eigenvalues.**

Finite-size effects, unavoidable in lab experiments, can significantly alter the turbulent behavior of nonlinear waves predicted by weak wave turbulence theory. Depending on the wave nonlinearity and container geometry, two regimes can be distinguished: Discrete wave turbulence, where exact wave resonances (e.g., 3-wave, 4-wave, etc.) dominate energy cascades, and kinetic wave turbulence, where statistical mixing prevents exact resonances under multiple scattering, causing deviations from canonical WT-theory. In Ref. [15], this distinction is based on comparing the nonlinear interaction rate, $\tau_{NL}^{-1}$, to the frequency spacing of the container eigenmodes, defined as a spectral broadening: $\Delta_{\omega}\approx\frac{2\pi}{L}\frac{d\omega_{k}}{dk}$.

In the case of nondispersive acoustic RWs, $\omega_{RW}\approx{(G/\rho)}^{1/2}k$, we deduce scaling dependencies $\left. \tau_{NL}^{-1} \right|_{RW} \approx(\rho/G)A^{2}{(\omega/\omega_{0})}^{-7/2}\omega^{3}$, and $\Delta_{\omega}\approx\left( {2\pi}/L \right)\left( {4G}/\rho\right)^{1/2}$, with $\omega_{0}$ being the driving frequency and $L=15cm$ the diameter of the vessel. Based on the dimensional arguments presented in Ref. [15] in the main text, the statistical turbulence regime occurs when ${\tau_{NL}^{-1}\gg\Delta}_{\omega}$ (instantaneous interactions compared to instrumental broadening). Since $\tau_{NL}^{-1}$ has a direct dependence on the wave amplitude, $A$, it is expected that this condition is fulfilled at high wave excitations. Contrarily, when ${\tau_{NL}^{-1}\ll\Delta}_{\omega}$ (quasi-static exact resonances), discretization effects cannot be neglected, and the discrete wave turbulence regime is the dominant one. The compared analysis of these dynamic regimes appears in Suppl. Fig. SN3-1.


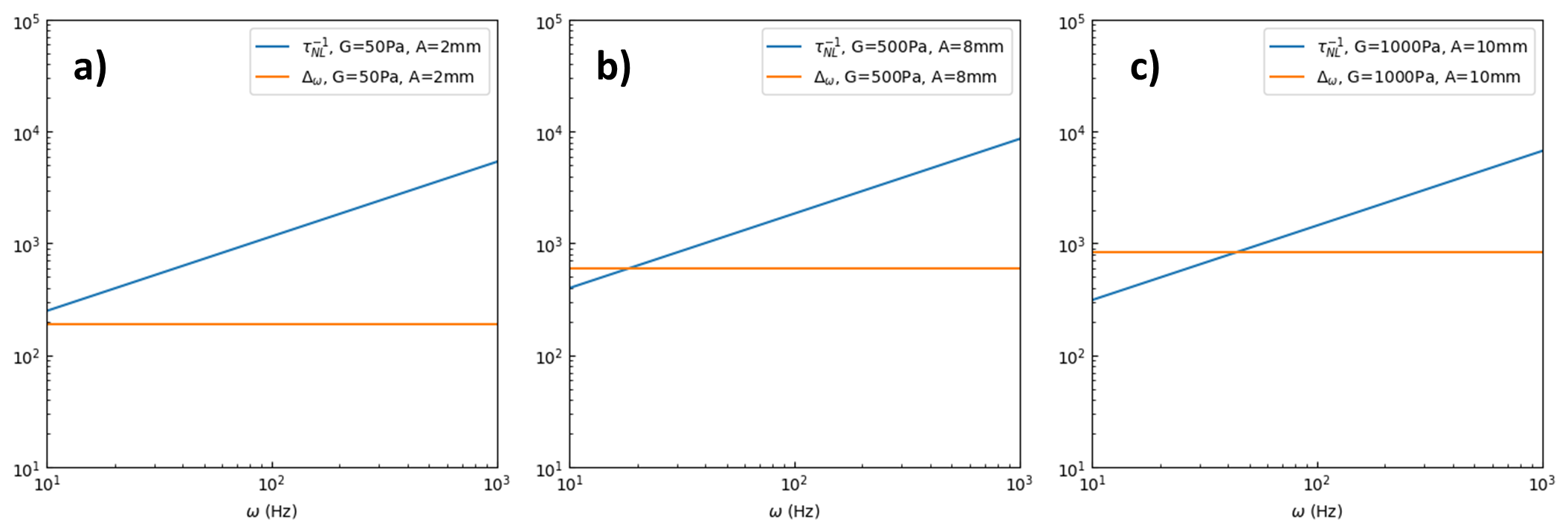


**Figure SN3-1. Nonlinear interaction rate** $\left. \tau_{NL}^{-1} \right|_{RW} \approx(\rho/G)A^{2}{(\omega/\omega_{0})}^{-7/2}\omega^{3}$ ***versus* dynamic spacing of vessel eigenvalues expressed as spectral broadening** $\Delta_{\omega}\approx2\pi/L\left( 4G/\rho\right)^{1/2}$ . Left panel: $G=50Pa$ with waves excited at $A=2mm$, Center panel: $G=0.5kPa$ with $A=8mm$, and Right panel: $G=1000Pa$ with $A=10mm$. Common excitation frequency: $\omega_{0}=50Hz$. Common spectral span range of the inertial interval: $\omega=10-1000Hz$.

To ensure the kinetic turbulence regime under discreteness conditions for weakly nonlinear RW turbulence in our experiments ($\tau_{NL}^{-1}\approx0\ll\Delta_{\omega}$), we set a nonlinear coupling rate much larger than limiting line narrowing characteristic of elastic modes with dispersionless propagation under energy conservation (3-waves coupling with a quasi-static interaction rate $\tau_{NL}^{-1}\ll\Delta_{\omega}$). Figure SN3-1 illustrates three cases (for $G=50Pa$, $G=0.5kPa$, and $G=1.0kPa$), over a typical frequency range ($\omega=10-1000Hz$), and typical values of $A$ used in experiments. In each case, the nonlinear interaction rate significantly vanishes with respect the spectral narrowing $(\Delta_{\omega}\approx0$), confirming that our results in this paper primarily reflect the turbulence regime under discreteness and exactness conditions. Calculations are based on $\omega_{0}=50Hz$ (as discussed in the main text). As bulk rigidity increases, the necessary excitation frequency to preserve discrete effects also rises.

**Supplementary Figures S1-S10**

**Supplementary Figure S1: Agarose hydrogel. Constitutive relation** $\boldsymbol{\tau=}\boldsymbol{G}^{\boldsymbol{*}}\boldsymbol{\gamma}$**,** $\boldsymbol{G}^{\boldsymbol{*}}\boldsymbol{=G+iG´´.}$ **Left panel:** variation of the stress $\tau$ with amplitude $\gamma$ of deformation for three different agarose concentrations, with $G=6Pa$, $80Pa$ and $500Pa$. Linear and nonlinear domains are clearly distinguishable. **Right panel:** variation of $G^{*}$in the same range of deformations, $\gamma$**;** note that in the linear regime $G^{*}$ is constant; the real value of the corresponding viscosity, $\eta$ and bulk rigidity, $G$, are obtained dividing $G^{*}$ by shear frequency, which in this case is $\omega_{s}=1Hz$.

**SUPPLEMENTARY FIGURE S2: Agarose hydrogel dynamic rheology. Upper panels: A)** *storage* modulus and **B)** *loss* modulus at different bulk rigidity values (plateau values of SUPPLEMENTARY FIGURE S1, right panel): $G=6Pa$, $G=50Pa$, $G=500Pa$ and $G=1700Pa$; both moduli are independent of shear frequency in a wide range ($1-100Hz$). **Lower panels: C)** phase angle, $\phi$ of the rheological measurement with values close to 0º, illustrating predominant elastic behavior, except for $G=6Pa$, for which an abrupt deviation at right $90º$ angle occurs indicating transition from solid-like to liquid-like behavior, around $10Hz$. **D)** complex modulus variation with agarose monomer concentration. The higher the concentration, the harder the hydrogel, where $G$ always predominates over$G´´$. Values taken at $\omega_{s}=1Hz$shear frequency and $1\%$ shear amplitude. Straight lines represent best fits to phenomenological power-laws describing agarose gelation under physical entanglement, $G\sim G^{'}\sim\left( c-c_{crit} \right)^{\theta}$, under low gel point, $c_{0}\approx0$, with scaling exponent $\theta\approx3$.

**SUPPLEMENTARY FIGURE S3: Rheological characterization of different materials (hydrogels, [oil/water] emulsions, and solidlike foams).** We can observe the predominance of *storage* modulus $G$ (black dots), over *loss* modulus, $G´´$(open circles), depicting a predominantly elastic behavior for all type of materials analyzed in this study.


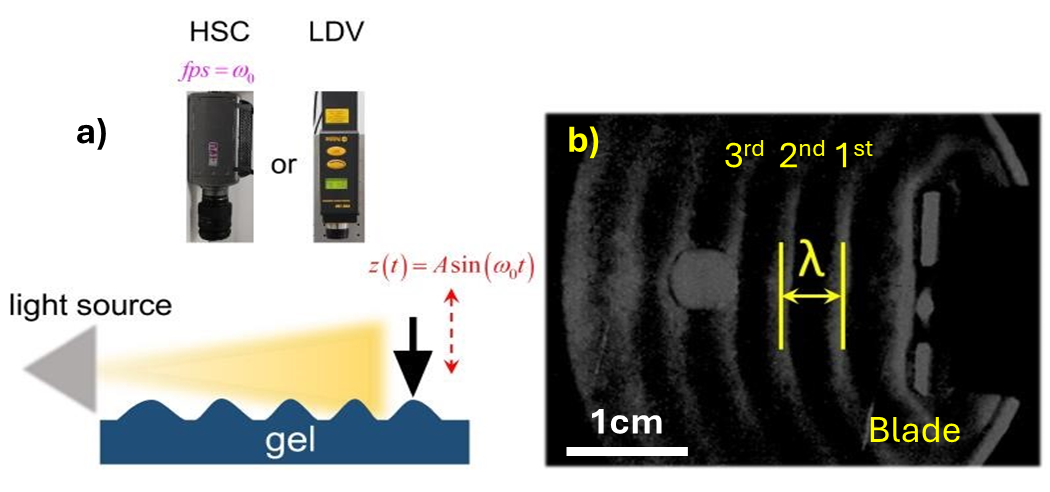


**SUPPLEMENTARY FIGURE S4. Experimental set-up used to characterize surface waves**. **A)** For the wave phase measurement, a High-Speed Camera (HSC) is used with a horizontally tilted white light source. Determination of the wave front separation is done by recording with a number of frames per second equals to the frequency of excitation: $fps=\omega_{0}$. For the nonlinear spectrum corresponding to the WT, the LDV laser is pointed to a stable central point of the vessel to avoid wave distortion due to reflection at the walls and each spectrum of turbulence is recorded as an average over 100 measurements across the entire acoustic range of frequencies, from $0-20kHz$. **B)** Top view of the vessel filled with hydrogel (Agarose, $G=50Pa$); waves are excited transversally with a blade and propagate from right to left. The successive wave fronts are indicated as 1^st^, 2^nd^, 3^rd^ and so on.

**SUPPLEMENTARY FIGURE S5: Agarose hydrogel. Dispersion relationships. Wave number dependence of oscillation frequencies and wave phase speeds, as agarose concentration changes. Left panel:** dispersion relationship for $G=6Pa\ll G^{*}$ corresponding to pure CWs (black circles); $G^{*}=80Pa$ close to CW/RW crossover (red circles; see Fig.1b in the main text); $G=1000Pa$ corresponding to pure RWs (red squares). Crossover appears for $G^{*}=80Pa$, as a continuous transition from acoustic RW propagation, $\omega_{RW}\approx{(G/\rho)}^{1/2}k$ (at $k<k^{*}=G/\sigma\approx1500 m^{-1}$), up to dispersive CW propagation, $\omega_{CW}\approx{(\sigma/\rho)}^{1/2}k^{3/2}$ (at $k>k^{*}$). The straight lines correspond to theoretical predictions using systemic parameters ($G=6, 80, 100 Pa$, $\sigma=72 {mN}/m$ and $\rho={10}^{3} {kg}/{m^{3}}$). **Right panel:** phase velocities. The different results for the three values of bulk rigidity are better visualized in the different propagation regimes including the gravity wave regime at very short wavevector. In the RW regime, the higher $G$, the higher the phase velocity $c_{R}\sim{(G/\rho)}^{1/2}$.

**SUPPLEMENTARY FIGURE S6: Agarose hydrogels. Turbulence spectrum versus agarose concentration. Transition from CW to RW.** As mentioned in the main text, transition from CW to RW is expected either by changing the frequency of excitation (see main text, Fig.1) or by changing the concentration while exciting surface waves at the same frequency. As we increase the agarose concentration there is increase of $G$; at lower values of $G$ we have pure CW turbulence, slope $-17/6$, while at higher values of $G$ the slope changes to $-5/2$, in nice agreement with our analysis for RW (KZ) turbulence.

**SUPPLEMENTARY FIGURE S7: Maximum inertial interval Kolmogorov frequencies for higher range of concentrations in agarose hydrogels:** $G=500Pa$ (grey symbols) and $G=1500Pa$ (red symbols) where RW are excited at two different frequencies, $\omega_{0}=48Hz$ (solid circles) and $\omega_{0}=27Hz$ (open circles). Straight lines correspond to the theoretically derived power-law for RWs: $\omega_{max}\cong K_{RW}A^{4/5}\omega_{0}^{7/5}$, under constitutive amplitude parameter $K_{RW}\equiv{(\rho/2\eta)}^{2/5}$ (see Eq. 13 in main text). Systemic parameters: $\rho={10}^{3} {kg}/{m^{3}};\eta=20 Pa.s$(for $G=500Pa$; grey symbols); $\eta=50 Pa.s$(for $G=1500 Pa$; red symbols). Note that the *inertial interval* in reduced when increasing the value of $\eta\left( G \right)$, by increasing agarose concentration (see **SUPPL. FIG. S1** and **SUPPL. FIG S2**).

**SUPPLEMENTARY FIGURE S8: Agarose hydrogels. Spectral linewidth of the nonlinear inertial spectral peaks for hydrogels with increasing agarose concentration.** As stated in the main text, the spectral broadening becomes progressively lower with increasing concentration of agarose gels because of the relative increase of rigidity supporting exact resonances over viscosity, responsible of wave damping by friction. **Left panel:** results for three different concentrations of the agarose gel excited at $\omega_{0}=65Hz$ fitted with Lorentzian distributions centered around a common single spectral peak. Here, we display the first discrete harmonics for comparison in the lowest resonant mode. The concentration of the hydrogel increases from top to bottom **Right panel:** area integration of the spectral first-harmonic under decreasing broadening within the range of frequencies shown in the left figure. Note the significant reduction in spectral broadening by several orders of magnitude as the system transitions from liquid-like behavior at low agarose concentrations to solid-like behavior with increasing concentration.

**SUPPLEMENTARY FIGURE S9: Parental Primality of 3-wave “sister” frequencies in agarose hydrogel. *Top)* Sum-frequency Rayleigh wave (RW) turbulence spectrum from dichromatic excitation.** Notably, the spectrum exhibits a complex structure due to the superposition of three distinct wave cascades: those from the harmonics of both fundamental excitation frequencies ($\omega_{1}$ and $\omega_{2}$), and their 3-wave interaction sum ($\omega_{3}=\omega_{1}+\omega_{2}$). The black open circles mark the spectral peak positions, while the black line shows the nonlinear fit with a $-5/2$ characteristic of the Kolmogorov-Zakharov (KZ) turbulence spectrum for RW, as illustrated in the main text (Fig. 2). ***Bottom)* Higher harmonic analysis.** The panels display the harmonic counts for the three primary frequencies, showing only the harmonics directly corresponding to these sister frequencies ($\omega_{1}, \omega_{2}$ and $\omega_{3}$).

**SUPPLEMENTARY FIGURE S10: Inertial turbulence interval in solidlike dry foam. Left panel:** typical KZ-spectrum of discrete RW turbulence. The red line corresponds to KZ turbulence with a spectral slope $-5/2$. **Right panel:** maximum frequency $\omega_{max}$ against wave amplitude, $A$, providing a power-law RW dependence: $\omega_{max}\cong K_{RW}A^{4/5}\omega_{0}^{7/5}$ (see Eq. 13 in main text). The fundamental excitation frequency is $\omega_{0}=100Hz$. Straight red line corresponds to the theoretically derived power-law under constitutive amplitude parameter $K_{RW}\equiv{(\rho/2\eta)}^{2/5}$. Systemic parameters: $\rho={10}^{3} {kg}/{m^{3}};\eta=4 Pa.s$(for $G=80 Pa$). Experimental results show excellent agreement with predictions of the theory for RW generated on the surface of solid-like dry foam (see Suppl. Note SN3).
